# A high-purity gas–solid photoreactor for reliable and reproducible photocatalytic CO_2_ reduction measurements

**DOI:** 10.1016/j.ohx.2023.e00448

**Published:** 2023-06-28

**Authors:** Nikolaos G. Moustakas, Marcus Klahn, Bastian T. Mei, Anna Pougin, Martin Dilla, Tim Peppel, Simon Ristig, Jennifer Strunk

**Affiliations:** a Leibniz Institute for Catalysis (LIKAT), Albert-Einstein-Str. 29a, 18059 Rostock, Germany; b Ruhr University Bochum, Universitätsstr. 150, 44801 Bochum, Germany; c Max Planck Institute for Chemical Energy Conversion (MPI CEC), Stiftstr. 34-36, 45470 Mülheim an der Ruhr, Germany

**Keywords:** Photocatalytic CO_2_ reduction, High-purity conditions, Gas-phase photoreactor, Heterogeneous photocatalysis

## Abstract

Reactions between a gas phase and a solid material are of high importance in the study of alternative ways for energy conversion utilizing otherwise useless carbon dioxide (CO_2_). The photocatalytic CO_2_ reduction to hydrocarbon fuels like e.g., methane (CH_4_) is such a potential candidate process converting solar light into molecular bonds. In this work, the design, construction, and operation of a high-purity gas–solid photoreactor is described. The design aims at eliminating any unwanted carbon-containing impurities and leak points, ensuring the collection of reliable and reproducible data in photocatalytic CO_2_ reduction measurements. Apart from the hardware design, a detailed experimental procedure including gas analysis is presented, allowing newcomers in the field of gas–solid CO_2_ reduction to learn the essential basics and valuable tricks. By performing extensive blank measurements (with/without sample and/or light) the true performance of photocatalytic materials can be monitored, leading to the identification of trends and the proposal of possible mechanisms in CO_2_ photoreduction. The reproducibility of measurements between different versions of the here presented reactor on the ppm level is evidenced.

## Highlights

• A high purity photoreactor design for reliable CO_2_ photoreduction experiments.
• Design taking into consideration all sources of carbon containing impurities.
• High reproducibility of product yields through different iterations of the reactor.
• Reactor design and experimental process comply with DIN SPEC 91457.

## Graphical abstract

**Figure:**
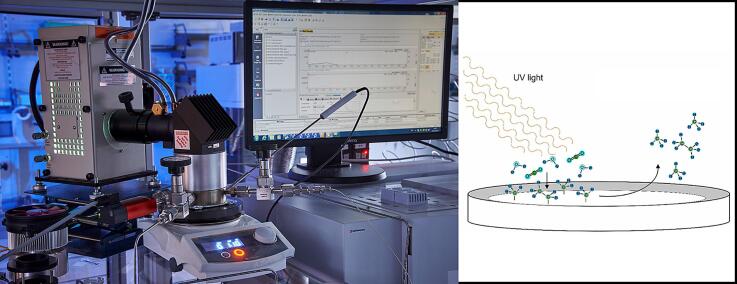

**Specifications table.**

**Table:** 

Hardware name	High-purity gas-phase photoreactor for reliable and reproducible photocatalytic CO_2_ reduction measurements.
Subject area	Chemistry and Biochemistry
Hardware type	*Photoreactor for gas*–*solid reactions*
Closest commercial analog	*No commercial analog is available*
Open Source License	*Creative Commons Attribution-ShareAlike 4.0 International License (CC BY-SA 4.0)*
Cost of Hardware	*Approximately: 38,000 € (euros) without gas analysis*
Source File Repository	*https://doi.org/10.5281/zenodo.7848585*

## 1 Hardware in context

As the atmospheric concentration of carbon dioxide (CO_2_) continues to increase (reaching 420 ppm in March 2023) [1], its effects on the climate become increasingly more evident. As the human energy needs also continue to grow, renewable energy sources and energy transformation processes should be employed to mitigate the harmful effects of the greenhouse gases on the global climate. Such an energy transformation process is the photocatalytic reduction of CO_2_ where solar energy is converted into chemical energy stored in the form of molecular bonds. Under irradiation and in the presence of light and water, CO_2_ is converted to hydrocarbons and value-added products with the aid of materials (photocatalysts) able to absorb the incident light and drive the conversion reaction. As this process mimics natural photosynthesis, it is often found in literature under the term "artificial photosynthesis".

Many excellent reviews on CO_2_ photoreduction exist in the scientific literature presenting the advances and challenges of the field [2], [3], [4], [5], [6], [7], [8]. The number of unique photoreactor designs specially used in heterogeneous CO_2_ photoreduction is rather limited in literature [9], [10]. The quartz fiber reactor by Wu *et al*. is one of these unique designs where the photocatalyst is coated onto the outer surface of optical fibers and operates based on the difference of the refraction index between the coating and the optical fiber [11], [12]. While this design allows the uniform radiation of the photocatalyst, it involves a coating step of fragile optical fibers which at the end of the experiment are difficult (or maybe not possible) to be reused with a new photocatalytic material. Another design involves the deposition of the photocatalyst on the walls of multichannel monoliths where the irradiation is performed using optical fibers running through the monolithic channels [13]. This design offers a highly tunable geometry but exhibits similar challenges as the optical fiber design: a coating step, an intricate assembly of the monolith-optical fiber system, a limited reusability of the monolith substrate and a challenging photocatalyst recovery.

When it comes to the deposition of a photocatalyst on a substrate, glass beads are an option [14]. The coated glass beads are inserted inside a quartz tube (in a packed-bed design) and held in place using glass wool. The selected gas mixture flows through the tube which is surrounded by (UV) lamps. The use of the quartz tube design presents two challenges: i) the perfect sealing of the quartz tubes to the gas-carrying system (presumably performed using Ultra-Torr connectors) and ii) the non-uniform irradiation of the glass beads located towards the center of the tube. In addition, the gas sampling is performed using a gas-tight syringe which might introduce a potential source of inconsistent sampling.

The design of Varghese *et al*. consists of a stainless-steel chamber with valves for evacuation and gas supply, and a side located septum allowing for gas sampling [15]. The authors have conducted various blank experiments, which have provided evidence that hydrocarbons were not formed (or formed at very low concentrations), in the absence of a photocatalyst or/and under dark conditions, hence the central round chamber design was adopted by us here. However, the use of an elastomeric septum for sampling purposes, which may be prone to leakage, together with the lack of constant pressure monitoring, represents a potential drawback of the design.

In the research field of photocatalytic CO_2_ reduction, performing experiments under conditions of the highest possible purity is of upmost importance. As CO_2_ is thermodynamically a very stable molecule (ΔG_f_°_CO2_ = −394 kJ mol^−1^) [16], any carbon-containing impurity present in a photoreactor is likely to react first, leading to the formation of carbonaceous products falsely attributed to the performance of the photocatalyst. In a previous work of ours, we reported that in many published studies blank experiments (e.g., light irradiation of the photocatalyst in absence of CO_2_) were not performed (or not mentioned), rendering the reported material efficiencies doubtful [3], [17]. Thus, to reach safe conclusions about a tested photocatalyst, a photoreactor constructed for and operated under high-purity conditions is needed. Reliable conclusions and reproducible results lead to useful insights which can eventually increase the still low – and industrially non-appealing – photocatalytic yields in hydrocarbon production from CO_2_ and help researchers to unravel the underlying CO_2_ photo-conversion mechanism [2], [3]. As it will become evident in the following paragraphs, even if the photoreactor described in this work is especially designed and constructed for CO_2_ photoreduction, it can also be used for the study of different (light and/or heat driven) gas-phase reactions with the application of slight modifications to it.

## 2 Hardware description

As aforementioned, performing CO_2_ photoreduction experiments under high-purity conditions is critical to draw useful conclusions on the conversion mechanism of CO_2_ and the overall efficiency of the tested photocatalysts. Such a photoreactor design is being described in detail in this work. This design is an evolution of a high-purity gas-phase photoreactor setup constructed and operated by our respective groups in the past [18]. This enhanced and optimized setup offers a simpler construction and a more space-conscious realization while keeping the high-purity status and the reproducibility of measurements. At this point, it is important to mention that a highly detailed description of the design, construction, and operation of the photoreactor has never been attempted in the past, although all three versions of the photoreactors have been already used in CO_2_ photoreduction studies, the results of which have been published in peer reviewed journals by the authors of this work [19], [20], [21], [22], [23], [24], [25]. The goal of this work is to offer an elaborate guide for the interested readers to be able to replicate the photoreactor in their respective labs. In this photoreactor, every potential source of impurities has been identified and taken into consideration, with extensive efforts applied to eliminate them. The potential sources of impurities related to the design, construction, and operation of the photoreactor include (among others) the usage of elastomeric sealing rings and grease-based sealants, oil and oil fumes from the operation of vacuum pumps, leak points present in the reactor setup allowing for atmospheric air to enter the reaction chamber, and improper handling of the materials and the reaction chamber. A detailed discussion on these sources and how they can be eliminated is offered later in this work.

The design and construction of the photoreactor presented here ensures a leak- and impurity-free operation during a gas-phase CO_2_ photoreduction experiment. As the requirements and the desired studies vary between potential users who wish to replicate the design of this photoreactor, experimental parameters such as the product identification method and the light irradiation source can be selected freely. While in principle any product identification method can be used, it is advised to select one that offers accurate identification of oxygen (O_2_) and high sensitivity to hydrocarbons (C_1+_). The operation guide provided below is a detailed description of the experimental protocols used by us in our studies in the field of CO_2_ photoreduction. Its photoconversion to CH_4_ is presented in Eq. (1), but the photoreactor can also be used (with minor modifications) for the study of other important gas–solid processes under the influence of light irradiation, such as the photo (reverse)-water–gas-shift (photo-(R)WGS, Eq. (2) and the photo-dry reforming of CH_4_ (photo-DRM, Eq. (3)). Usually these gas-phase reactions run at high temperatures, but under light irradiation and in the presence of a suitable and stable photocatalyst, they have the potential to run at less harsh conditions.

(1) $$CO_{2}+2H_{2}O\to CH_{4}+2O_{2}$$

(2) $$\mathrm{CO}+H_{2}O⇌CO_{2}+H_{2}$$

(3) $$CO_{2}+CH_{4}⇌2H_{2}+2CO$$

## 3 Design files

A detailed schematic of the photoreactor and its interconnected parts is presented in Fig. 2. The engineering schematics with the exact dimensions including cross-sections of the custom-made parts of the photoreactor setup (water saturators and the main body of the reaction chamber) are included in the Supplementary Information of this manuscript and in https://doi.org/10.5281/zenodo.7848585.

**Fig. 1:**
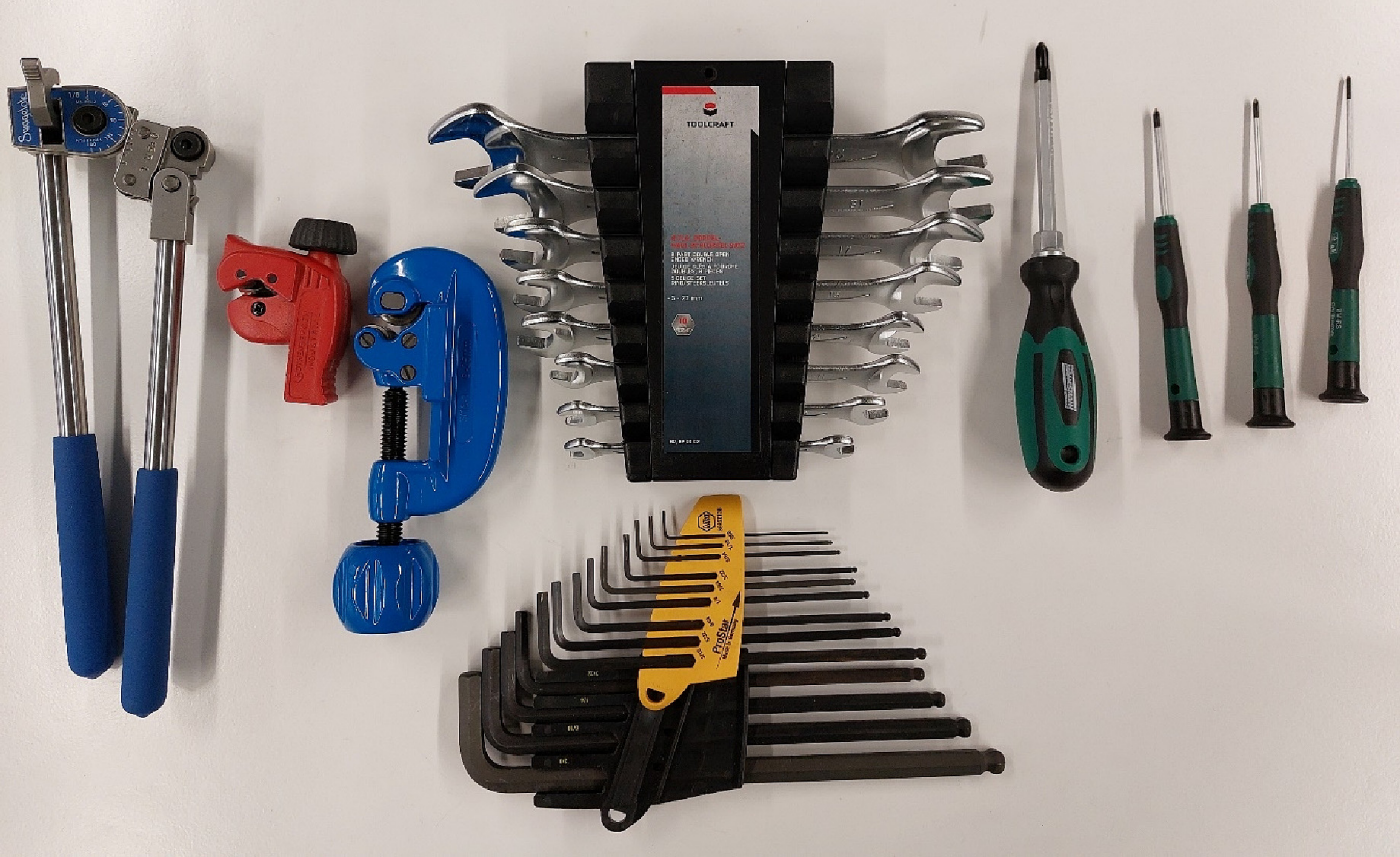
Hand tools required for the assembly of the high-purity photoreactor.

**Fig. 2:**
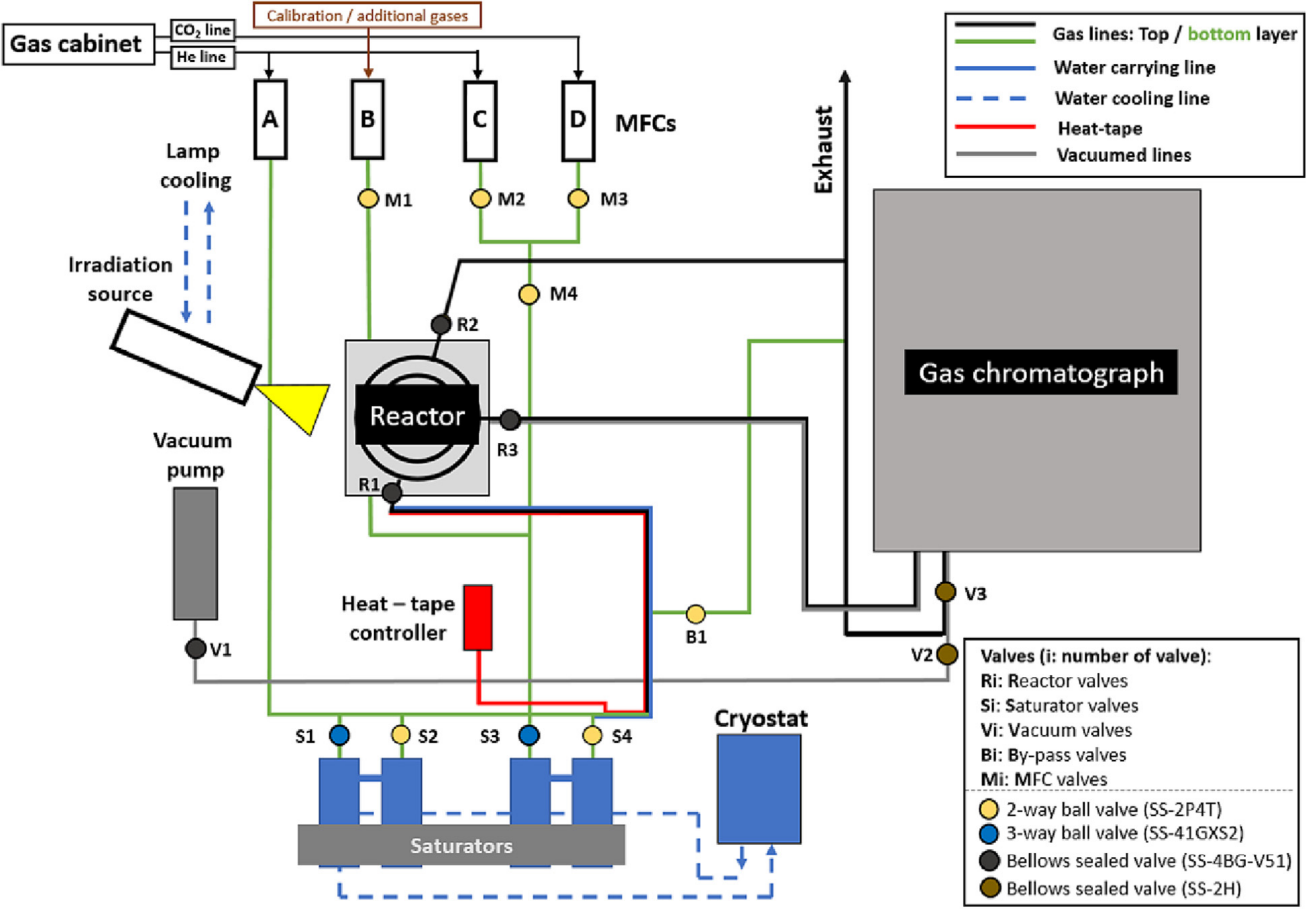
Schematic of the high-purity photoreactor system.

## 4 Bill of materials

A detailed Bill of Materials can be found in https://doi.org/10.5281/zenodo.7848585. It should be noted that the cost of the product identification device (e.g., gas chromatograph, mass spectrometer) is not included as its price depends on the requirements of the reaction(s) that the potential user is interested in. The same applies to the gas cylinders used for the experiments of interest and for the operation of the selected product identification method. The prices of gas cylinders of high purity gases (e.g., 6.0 He) vary significantly, heavily influenced by external factors increasing the operational cost of the device.

The high-purity photoreactor described in this work is the result of an optimization process over many years. Thus, it is possible that some of the prices included in the Bill of Materials have changed. Special effort has been paid by the authors to provide the reader with up-to-date prices wherever possible. It is very important to note that the prices included in the Bill of Materials refer to prices in Germany and it is very likely that significant changes in prices might occur depending on the region of the world (e.g., different delivery costs).

## 5 Build instructions

### 5.1 Hand tools required

The connection of the different main parts of the high-purity photoreactor is performed using stainless steel tubes (Swagelok, Solon, Ohio, USA) of different lengths and diameters, and various connectors, adaptors, and valves. All tube connections should be performed following the guidelines offered by the manufacturer to allow a leak-free assembly. The following tools were used for the assembly of the photoreactor: a hand tube bender (MS-HTB-4, Swagelok), a cutter for stainless steel tubes (MS-TC-308, Swagelok), a tube deburring tool (MS-TDT-24, Swagelok), a set of open-end wrenches, a set of Hex Keys / Allen wrenches and Phillips screwdrivers of different sizes (Fig. 1).

### 5.2 General description of the individual parts of the photoreactor

For a better comprehension of the photoreactor design by the reader, the description will be separated into four sections: i) the gas and water carrying system, ii) the reaction chamber, iii) the irradiation sources and iv) the product detection system. A schematic representation of the high-purity photoreactor is presented in Figs. 2 and S1 (Supplementary Information and https://doi.org/10.5281/zenodo.7848585). It is worth noting in advance that all parts of the photoreactor are made of stainless steel and are suitable for high-vacuum applications. The sealing and the connection of all interconnected parts are either performed using metallic O-rings or with stainless steel adaptors and connectors, allowing for a metal-to-metal seal without the need of any lubrication (grease-free sealing). This ensures a perfect seal (no inwards or outwards gas exchange) and that no impurities are introduced to the reactor from the use of elastomeric sealing rings. More details on the sealing materials and the used adaptors and connectors are offered in the following paragraphs.

#### 5.2.1 Gas and water carrying system

**Fig. 3:**
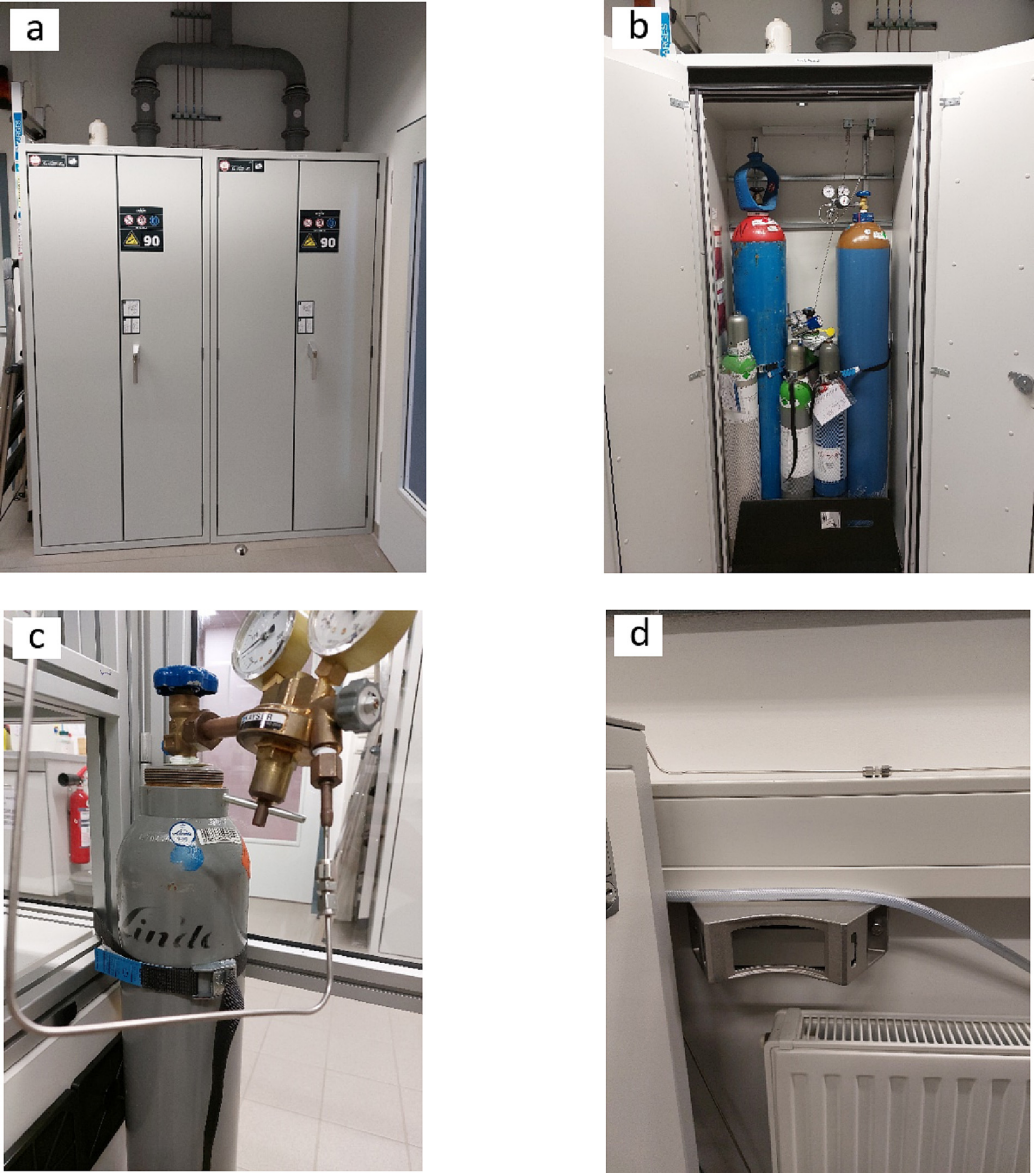
Ways of safe storage and placement of gas cylinders: a,b) Gas cabinet, c) metal rack holding and d) secured wall brackets.

All gas cylinders containing the required reactant gases should be stored and operated according to national regulations in the respective country in which the photoreactor is supposed to be operated. Taking Germany as an example, gas cylinders should best be located inside secure designated cabinets (Fig. 3a and b). If such cabinets are not available, the gas cylinder(s) should be mounted either on the metallic rack supporting the photoreactor (Fig. 3c) or by using special holding brackets securely attached to a wall (Fig. 3d).

**Fig. 4:**
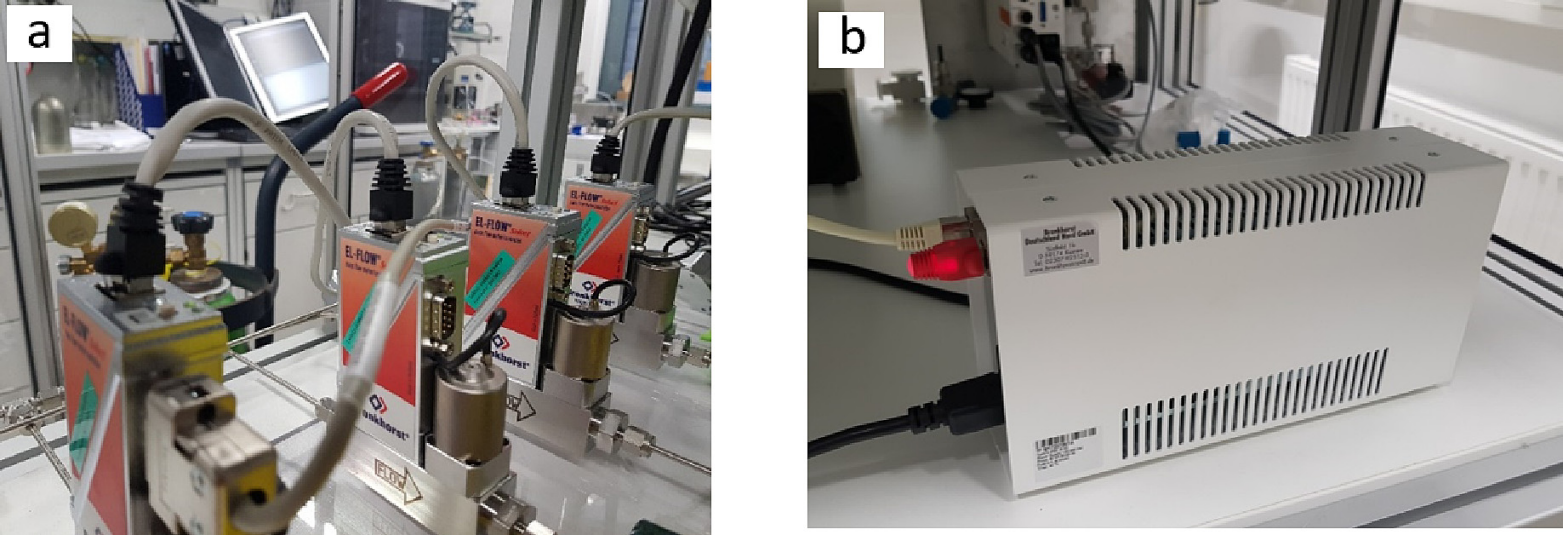
a) Mass flow controllers (MFCs) interconnected with Y-adapter cables and b) the E-8501R-00 power supply.

The two gas cylinders regularly used in the CO_2_ photoreduction experiments (pure 6.0 He and 4.8 CO_2_) are equipped with high-purity gas regulators (VIGOUR-1EC-300–10-00-P-P-00-R-B) not allowing ambient air diffusing inside the gas cylinders contaminating them and leaking of the reactant gases to the lab atmosphere. The pressure in both pressure regulators was set to 4 bar. The selected reactant gas(-es) from the cabinet is (are) guided through 1/8-inch stainless steel pipes (Swagelok) towards the photoreactor setup. The reactant gases are fed to the reaction chamber using four mass flow controllers (MFCs, EL-FLOW Select, Bronkhorst, Fig. 2, Fig. 4). The MFCs are electronically interconnected using Y-adapter cables (Bronkhorst) and all are powered by the E-8501R-00 power supply (Bronkhorst).

In the case of a typical CO_2_ photoreduction experiment MFCs C and D (see Fig. 2) supply pure He (6.0 purity, Linde) and CO_2_ (4.8 purity, Linde), respectively. The two remaining MFCs are used for gases (or gas mixtures) used for calibration purposes (MFC B) and for special gases added to the reaction mixture like H_2,_ CH_4_ or CO (MFC A). The MFCs are PC controlled using the FlowDDE and FlowView software (freely available to download from Bronkhorst). The gas flow through the MFCs can be controlled using as an input value either the actual preferred flow rate (in ml min^−1^) or a percentage of the maximum nominal flow rate of the MFC.

**Fig. 5:**
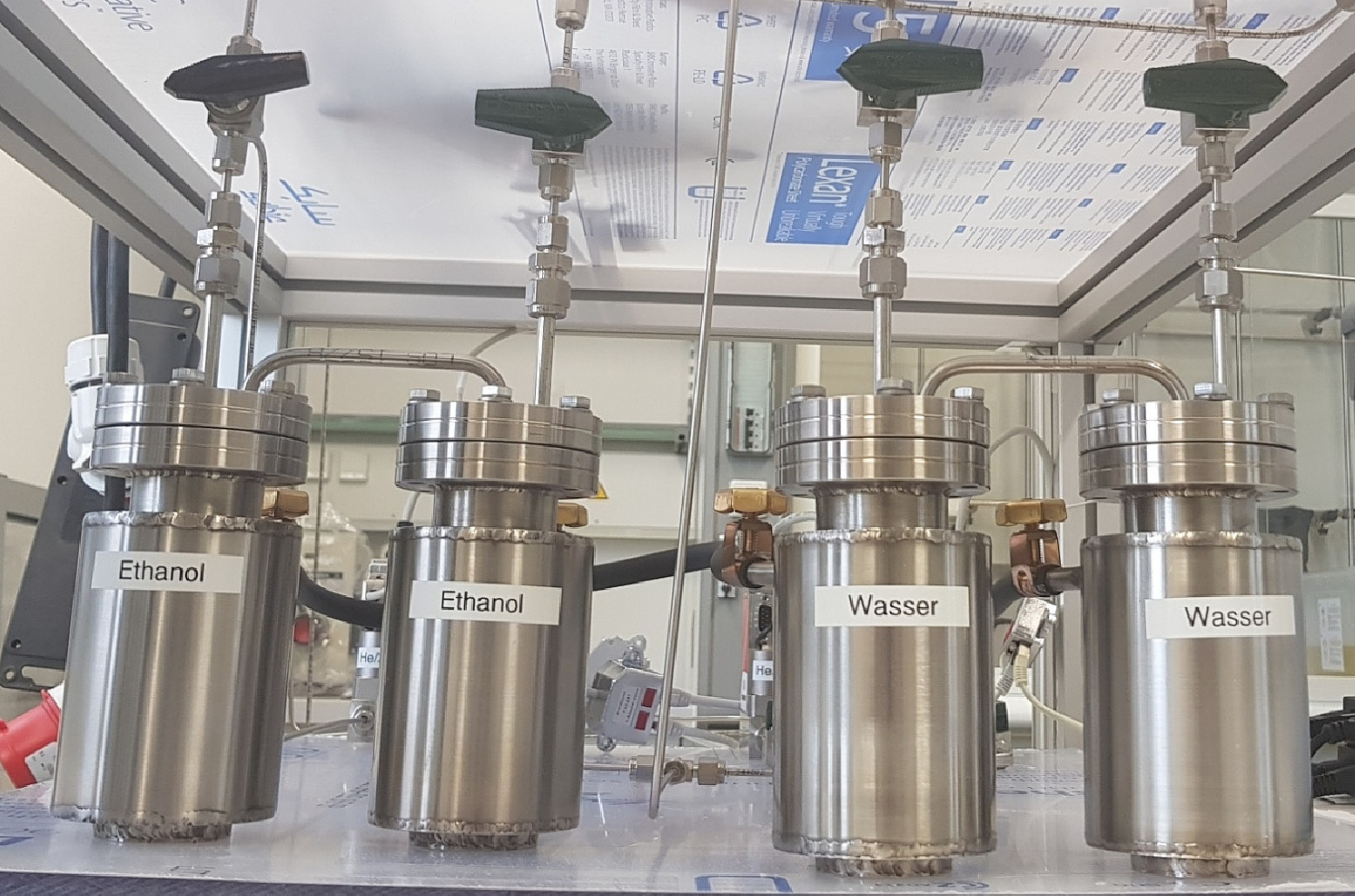
Two sets of saturators used for H_2_O (right pair) and other liquids (e.g., EtOH) (left pair).

In a typical photocatalytic CO_2_ reduction experiment the presence of H_2_O is required. For this reason, the MFCs are connected to two sets of saturators (Fig. 5). These saturators were designed and constructed in-house (LIKAT). A double-saturator design is chosen for the better equilibration of the saturation equilibrium of the liquid inside the gas flow. The detailed engineering design and the various views of the saturators are included in the SI (Figs. S2–S7, Supplementary Information and https://doi.org/10.5281/zenodo.7848585). From the two sets of saturators presented in Fig. 5, the set on the right is filled with high-purity water (ROTISOLV Pestilyse, Carl Roth) while the set of saturators on the left can be filled with other liquid reactants (e.g., ethanol or methanol) if an experiment requires so. The saturators feature a double-wall design (Fig. S2, Supplementary Information and https://doi.org/10.5281/zenodo.7848585) which allows the control of their temperature using a cryostat (Huber, Pilot ONE ministat 125, temperature range 5–90 °C). The cooled (or heated) water from the cryostat is circulating through the saturators using ROTILABO EX14.1 (Carl Roth) cooling tubes. The H_2_O concentration in the reactant gas stream can be calculated by the set temperature of the saturators using the Antoine equation (Eq. (4)) and the three temperature-dependent dimensionless parameters A, B and C (Table 1).

(4) $$P=10^{A-\frac{B}{T+C}}$$

**Table 1:** Dimensionless parameters of Antoine equation for temperature range 273–303 K.

A	B	C
5.40221	1838.675	−31.737

**Fig. 6:**
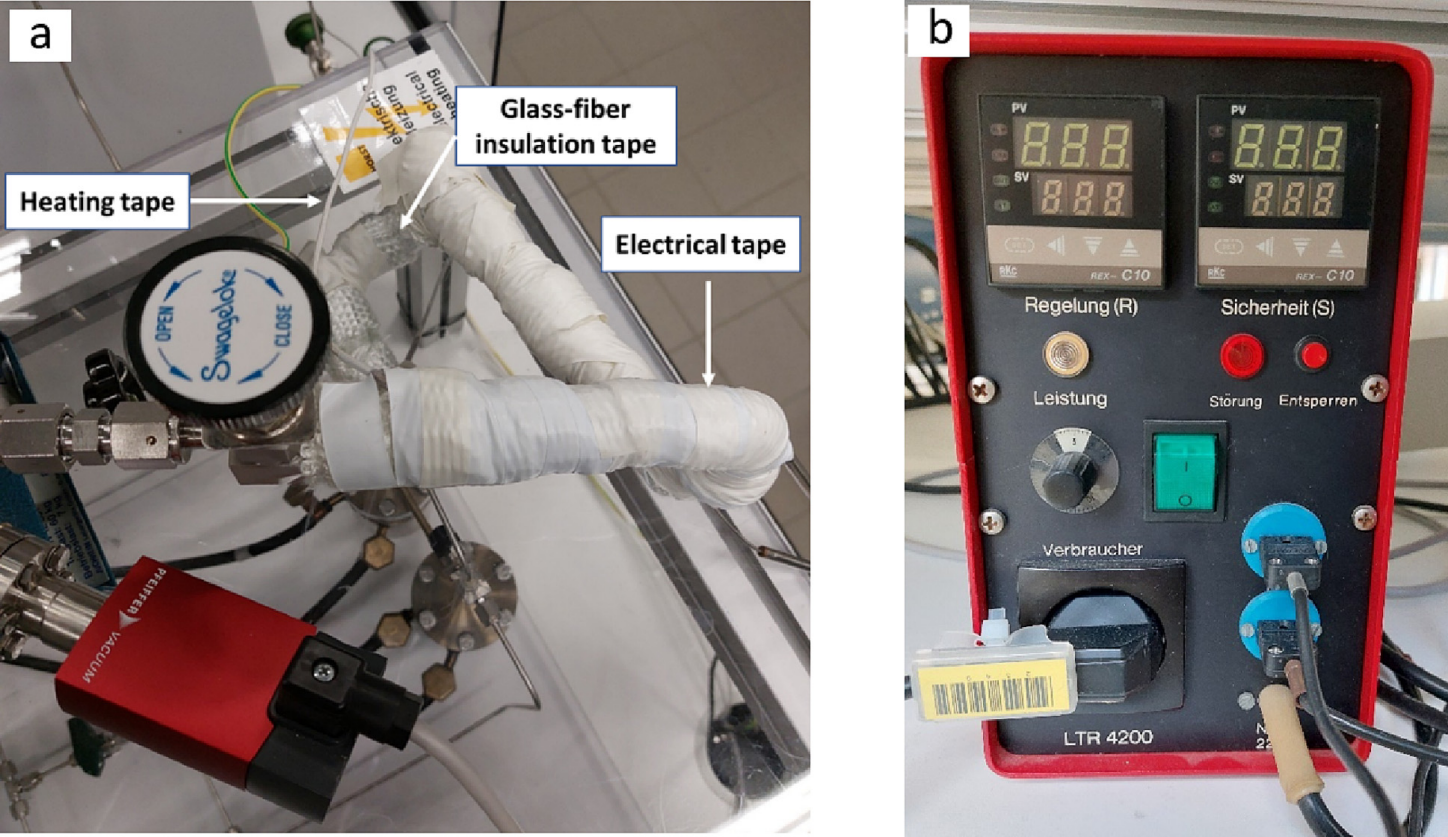
a) Insulated electrical heating tapes and b) LTR 420 power controller.

In the front side (outlet) of MFCs C and D two valves (Fig. 1, valves M2 and M3 respectively) are positioned (2-way stainless steel valves, SS-2P4T, Swagelok). These valves control the flow of He from MFC C (valve M2) or CO_2_ from MFC D (valve M3) through the gas lines (1/8-inch, Swagelok). When both M2 and M3 valves are in the open position, a mixture of CO_2_ and He can be selected by the respective flow settings of the MFCs leading to reactant gas compositions with variable CO_2_ content. Valve M4 is used to isolate MFCs C and D when the gas supply is performed by the other two MFCs (A and B). Valve M1 (SS-2P4T, Swagelok), located in front of MFC B controls the flow of gases used for calibration purposes or for additional gases needed for the studied reaction. The gas from MFC B can also flow through the water saturator if required. Mass flow controller A (MFC A) is directly connected to the second set of saturators which can be optionally used for liquid reactants other than H_2_O, such as methanol or ethanol (often used as sacrificial reagents). All H_2_O-carrying pipes (blue-colored lines in Fig. 2) are covered by heating tapes (Horst GmbH) operating constantly at a temperature of 120 °C to reduce H_2_O condensation effects (pipes covered with the heating tapes are red-colored in Fig. 2). The heating tapes are covered by a glass-fiber insulation band (100201, Horst GmbH) and further covered by an insulation (electrical) tape to avoid contact of the glass-fiber with the user of the reactor (Fig. 6a). The heating tapes are powered by an LTR 4200 (Juchheim, Solingen, Germany) temperature controller (Fig. 6b).

#### 5.2.2 The reaction chamber

The main body of the reactor is constructed out of stainless steel and schematics of its cross-sections are presented in Figs. S8–S10 (Supplementary Information and https://doi.org/10.5281/zenodo.7848585). The lid of the reactor is commercially available (VPCH42 - Ø 2,75 in. CF Flange, Uncoated UVFS Window, Thorlabs, Figs. S11-S12, Supplementary Information and https://doi.org/10.5281/zenodo.7848585) while the main body was designed and built in-house (LIKAT). The body features a double-wall construction, allowing the performance of experiments at different temperatures using e.g., water or (silicon) oil. To ensure that no oil from the manufacturing process was left on the surfaces of the reaction chamber leading to the formation of hydrocarbons under the influence of light, the main body of the reactor was heated up to 300 °C overnight before its first installation in the photoreactor setup. In addition, extensive blank experiments under light were performed and no C-containing products were identified (more details about the process can be found in paragraph 6).

**Fig. 7:**
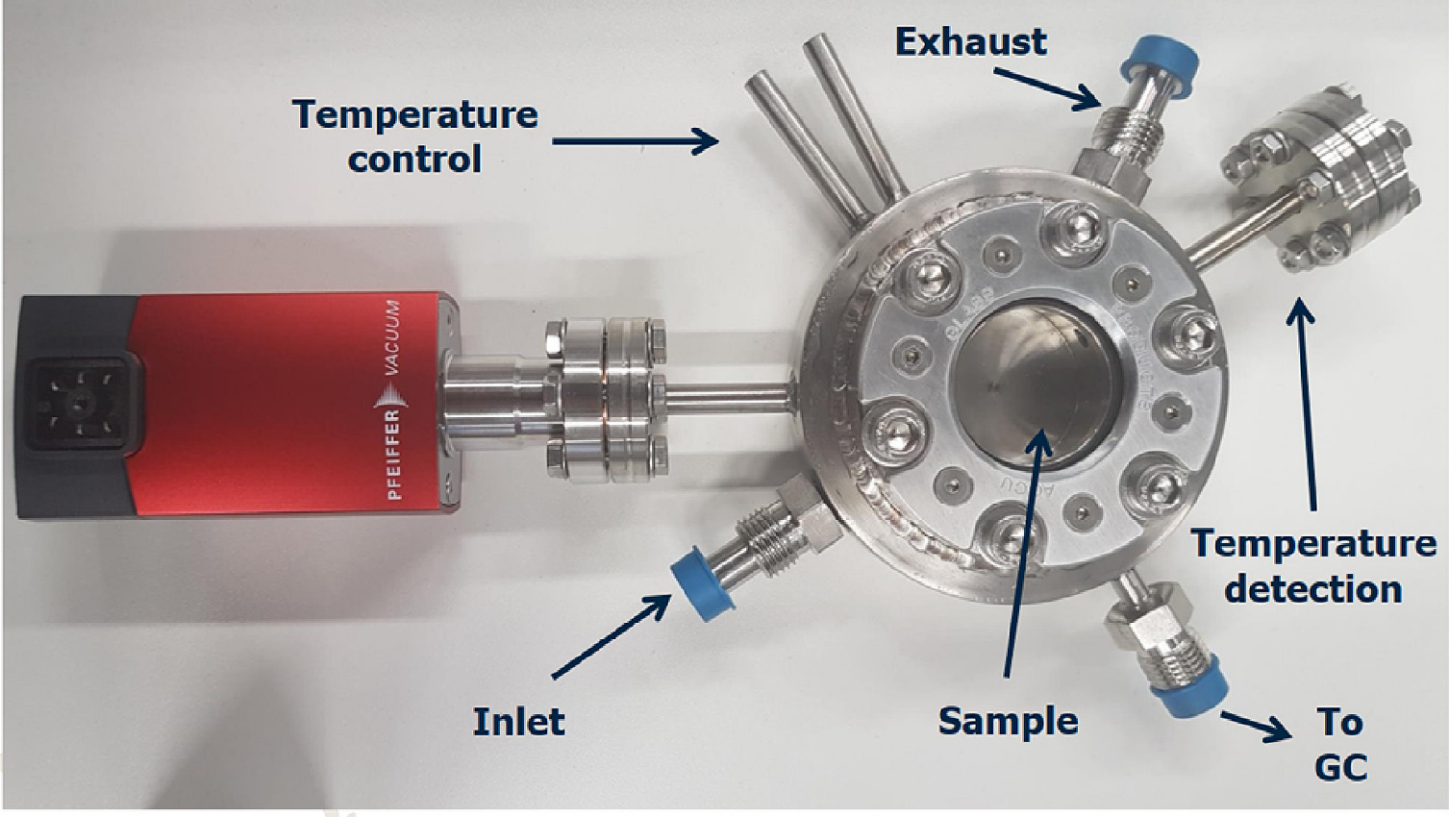
Close-up of the main body of the photoreactor.

**Fig. 8:**
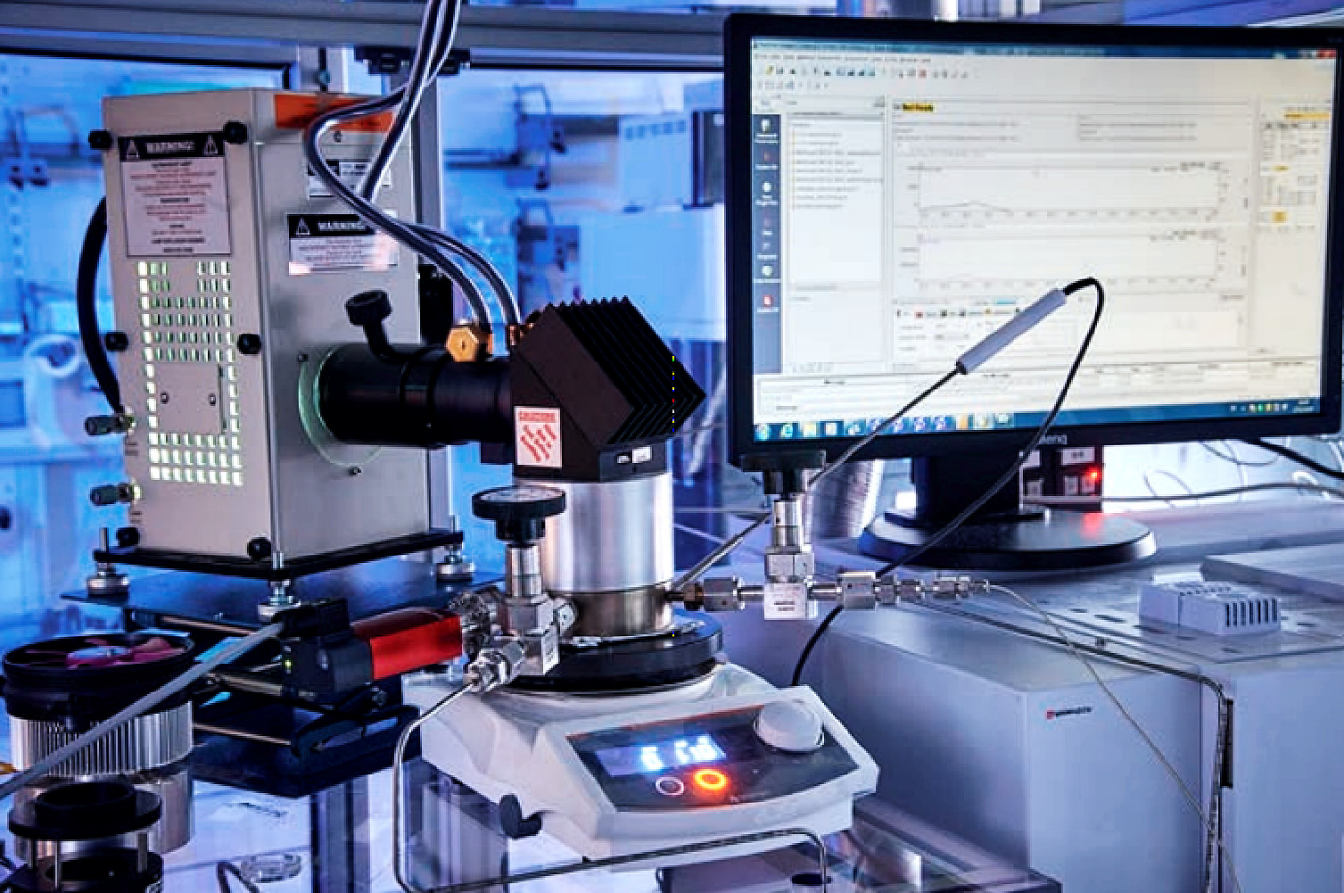
Main body of the photoreactor with the attached valves (R1-R2-R3) and pressure gauge meter. Below it, a heated stirring plate is placed. The main body is irradiated by a 200 W Hg/Xe lamp.

The lid is attached to the reactor using a set of six washers and bolts (A2-70 stainless-steel hexagon socket bolts, included with the lid). A close-up view of the reaction chamber can be seen in Fig. 7. The sealing of the reactor is performed by introducing a metallic O-ring (made of an O_2_-free high thermal conductivity copper coated with silver, OFC 40C, Vakuum-Anlagenbau GmbH) which is replaced every time the lid is removed from the main body of the reactor. This ensures a leak-free sealing of the reaction chamber. An additional heating option for the reaction chamber is the use of a heated stirring plate (MR Hei-Tec, Heidolph) located below the main body of the photoreactor (Fig. 8). This allows heating of the reactor up to 180 °C and/or the incorporation of liquid samples (maximum volume of 1 ml) in the reactor under constant stirring. The total volume of the reactor was estimated to be 30 ml (34.7 ml including the volume inside the pipes attached to the reaction chamber).

Three SS-4BG-V51 (Swagelok) valves are attached to the main body of the photoreactor. The selected (humidified) gas mixture enters the reaction chamber though the inlet port passing valve R1 (Fig. 1). In the reaction chamber the gas has three options: i) get released in the exhaust system of the lab through the exhaust outlet (through valve R2, Fig. 1), ii) pass through the sample loop (through valve R3, Fig. 1) of the product identification method (in our case a gas chromatograph – GC, more details in the following paragraphs), or iii) remain in the reaction chamber in a batch process (valves R1, R2 and R3 remain closed after filling the reactor to the desired pressure). All valves are connected to the main body of the reactor using 316L stainless steel VCR face seal fittings (SS-4-VCR-2-GR, Swagelok). A real-time depiction of the pressure inside the reaction chamber is achieved using a vacuum gauge meter (TPG 362, Pfeiffer) connected to the respective controller (PCR 280, Pfeiffer). The vacuum gauge meter is connected to the main body of the reactor using a DN 16 CF pipe connection (Fig. 7).

#### 5.2.3 Sample holders

**Fig. 9:**
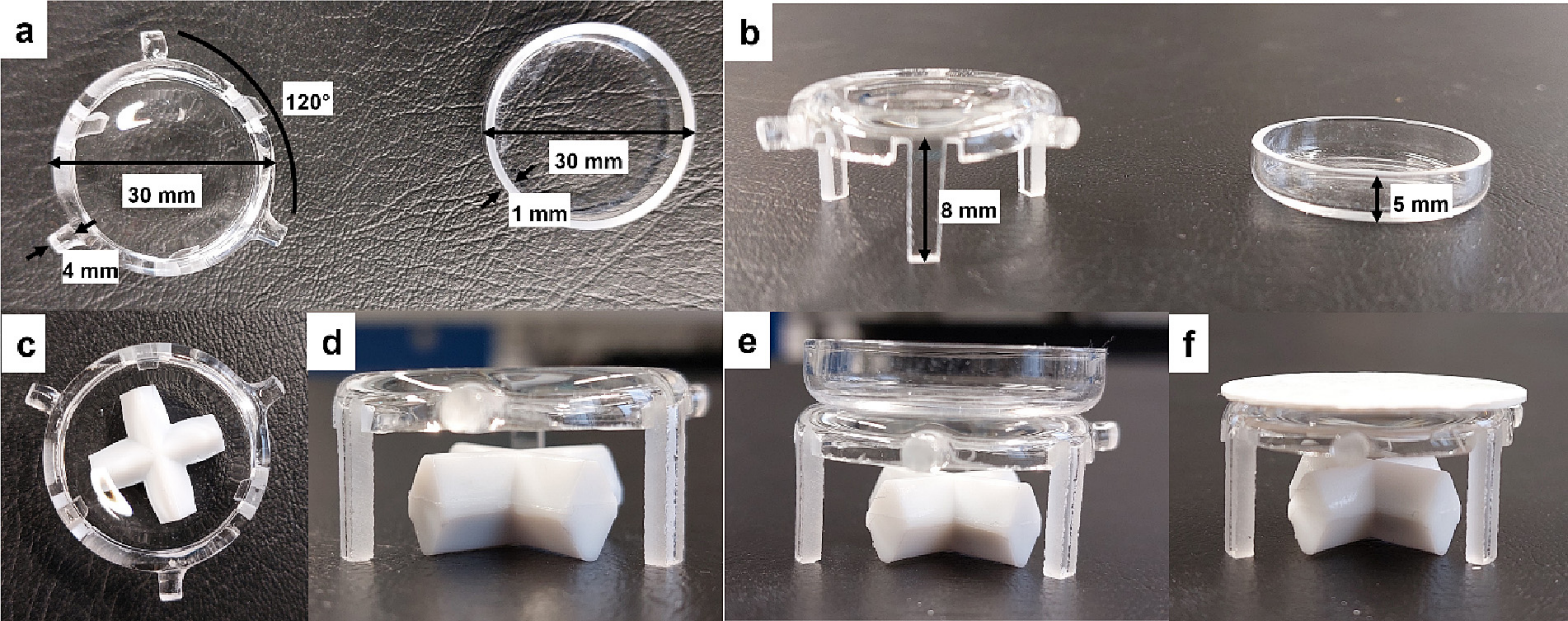
Different sample holders used in CO_2_ photoreduction experiments. a) top- and b) side- view of the raised sample holder and the quartz plate, c) top- and d) side- view of the raised sample holder with the stirring bar placed underneath. Side view of the raised sample holder with the e) quartz plate or f) a pellet of a photocatalyst placed on top.

With this reactor design, it is possible to use catalysts in the form of a powder, a pellet or deposited on an inert substrate (glass, ceramic etc.) as a thin film. When the catalyst is in a powder form, it is spread evenly inside a quartz sample holder (plate), see Fig. 9a and b. The sample holder is subsequently placed inside the reaction chamber. In the case of the deposited catalyst, the substate can be placed directly on the bottom of the reaction chamber. If there is a need to perform the experiment under circulation of the reactant gases inside the reaction chamber (e.g., in a batch experiment to increase the gas–solid contact time or/and to avoid concentration gradients) then the quartz plate or the deposited sample can sit on a specially designed holder which allows the rotation of a stirring bar below it (Fig. 9c–f). The stirring of the magnetic bar (PTFE, cross-shaped, Ø 20 mm, height 8 mm, XA22.1, Carl Roth) is being accomplished by the MR Hei-Tec heated stirring plate (Heidolph) (Fig. 8).

#### 5.2.4 Available irradiation systems

**Fig. 10:**
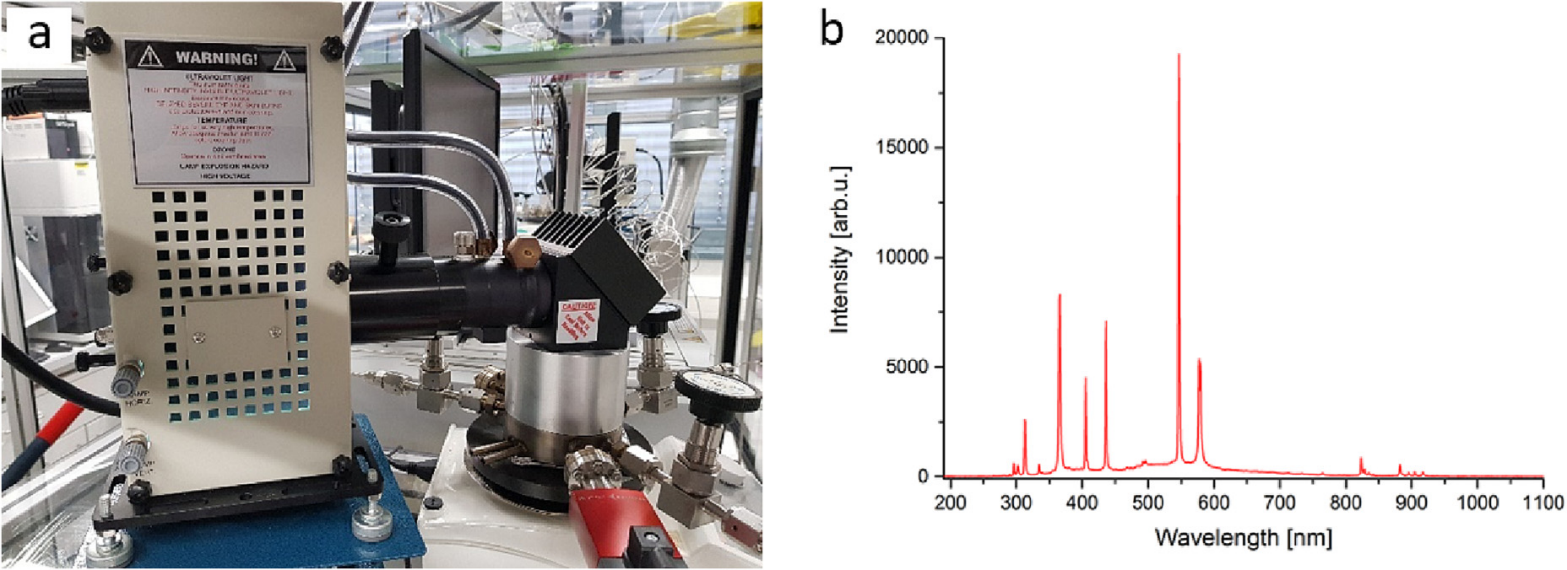
a) 200 W Hg/Xe lamp with the attached water-cooled IR filter and guiding mirrors and b) its irradiation spectrum.

The irradiation of the samples is realized through a 200 W Hg/He (Newport Oriel) lamp (Fig. 8, Fig. 10a) in a Newport 66,901 50–500 W lamp housing. The lamp is powered by an OPS-A500 (Newport) power unit. This irradiation system includes ultraviolet (UV), visible and near-infrared (IR) wavelengths (Fig. 10b) and its light intensity is set at 200mW cm^−2^. The light intensity is measured using an S405C measuring cell (Thorlabs) connected to a PM100USB (Thorlabs) power supply unit at a distance similar to the distance between the lamp and the sample when inside the photoreactor. A double-walled 1.5 in. water-filled filter (part number 61945, Newport) is used to absorb the IR part of the irradiation and to decrease the produced heat from the lamp avoiding an unwanted rise in the temperature inside the reaction chamber. This filter has a double-wall design in which cooled water (lab infrastructure) circulates to keep the water inside the filter at a constant temperature. The light beam is guided through a system of mirrors (part number 66245, Newport) to the top of the reactor and thus a large portion of the sample can be illuminated. Neutral density (ND) and cut-off filters can be placed in the light beam pathway allowing for e.g., the selection of a specific wavelength range.

**Fig. 11:**
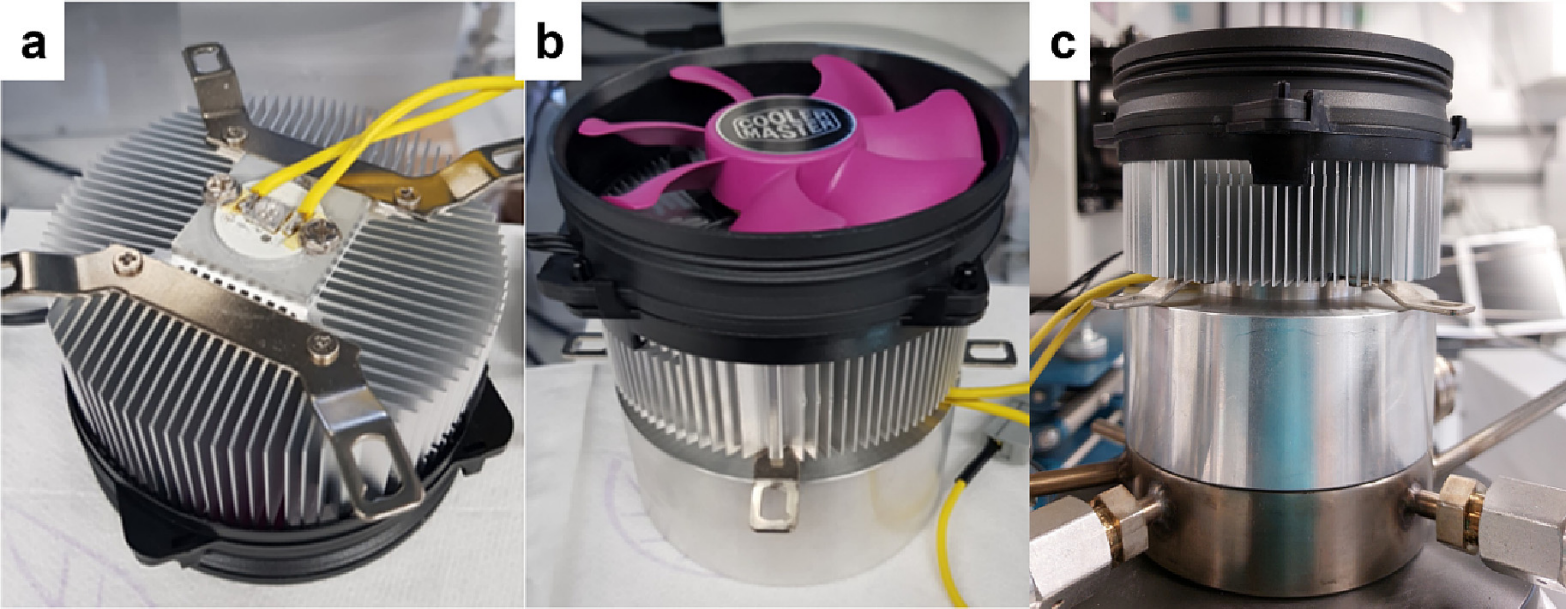
a) The high-power 365 nm LED (CUN6F4A), with b) its heat dissipation system and c) placed over the main body of the photoreactor.

As an additional light source, a high-power 365 nm LED (CUN6F4A, Neumüller Elektonik GmbH) is used in experiments where high-intensity UV irradiation is required (Fig. 11a). The LED is powered by a TOPS-3602 (VOLTCRAFT) power supply unit and operated at 1.0 A and 7.2 V. As the heat generated while the LED operates is very high, an efficient heat dissipation system (A200 performance C series, Xilence) is installed over the LED to ensure its safe operation (Fig. 11b). A thermal paste was placed between the heat dissipation system and the LED to increase the contact area and thus the cooling rate. As in the Hg/Xe lamp, the irradiation of the sample with the LED is performed from the top side of the reaction chamber (Fig. 11c).

**Fig. 12:**
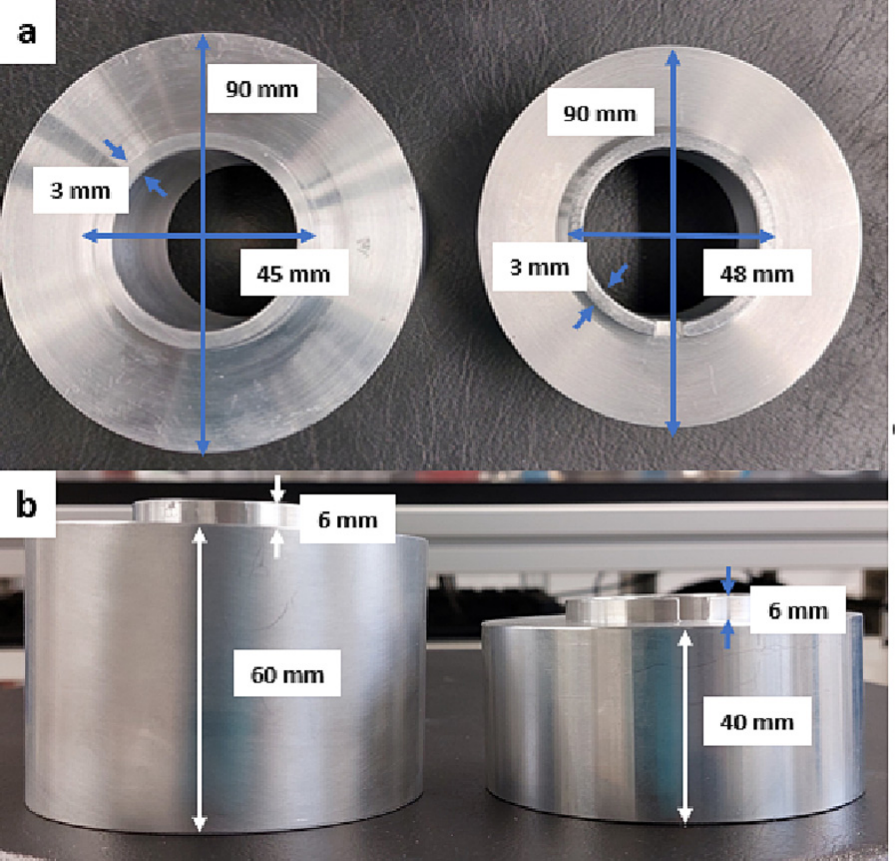
a) Top and b) side view of the aluminum spacers used with the Hg/Xe lamp (left) and the high-power LED (right).

To ensure that no light escapes and to keep a constant distance from the sample when operating the irradiation systems, aluminum spacers were constructed (in-house, LIKAT) (Fig. 12). In the case of the Hg/Xe lamp the spacer is safely attached to the end of the light guiding mirror system using 3 stainless steel M3 × 4 mm hex allen head screws.

The narrow distribution of irradiation wavelengths in the case of the LED allows for the calculation of the apparent quantum yields (AQY%) of the tested photoreactions. The importance of such a measure is of significance as product formation can be affected by the intensity of the light source used in a photocatalytic experiment. The apparent quantum yield, which is the ratio between the consumed charge carriers and the incident photons, can be determined by measuring the light source intensity of a monochromatic light. This approach is commonly used to avoid the issue of quantifying incident, reflected, and scattered photons (a rather challenging process, necessary though for the calculation of the quantum yield – QY) and instead relies on determining only the amount of incident photons. A detailed example of the calculation of the AQY% of experiments performed in the photoreactor described in this manuscript is presented in a previous work of our groups [22].

#### 5.2.5 Product identification: Gas chromatograph

**Fig. 13:**
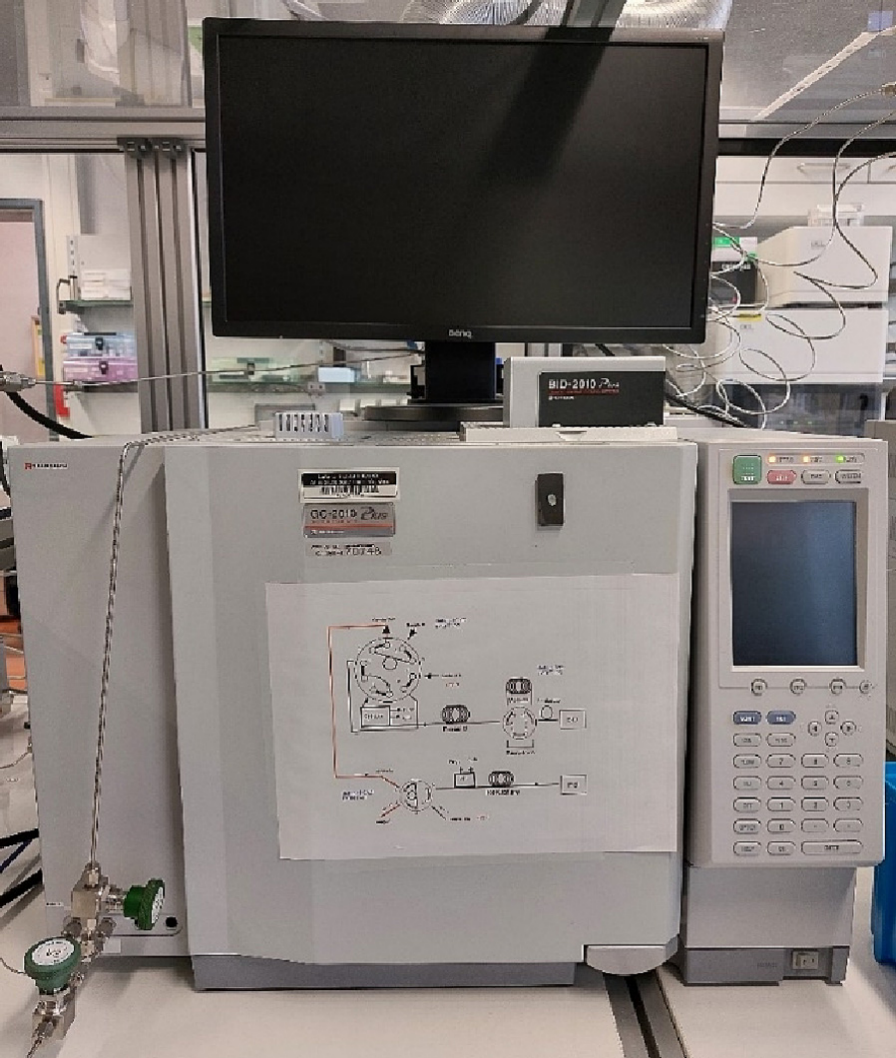
Tracera-2010 gas chromatograph equipped with an FID and a BID detector.

The identification of the gaseous products of the photocatalytic CO_2_ reduction experiments is performed using a gas chromatograph (TRACERA-2010, Shimadzu) equipped with a flame ionization detector (FID) and a barrier discharge ionization detector (BID) (Fig. 13). This combination of detectors allows the identification and quantification of reactants and products (H_2_O, CO_2_, O_2_, N_2_, and C_1_-C_14_ hydrocarbons) down to the 1 ppm range. For H_2_ and CO the limit was identified to be 20 ppm at atmospheric pressure. The outlet of the reactor is directly connected to the GC (through valve R3, Fig. 2) ensuring that there is no contact between the reacting gases and ambient atmosphere. The sampling is done automatically to avoid any user-induced errors.

**Fig. 14:**
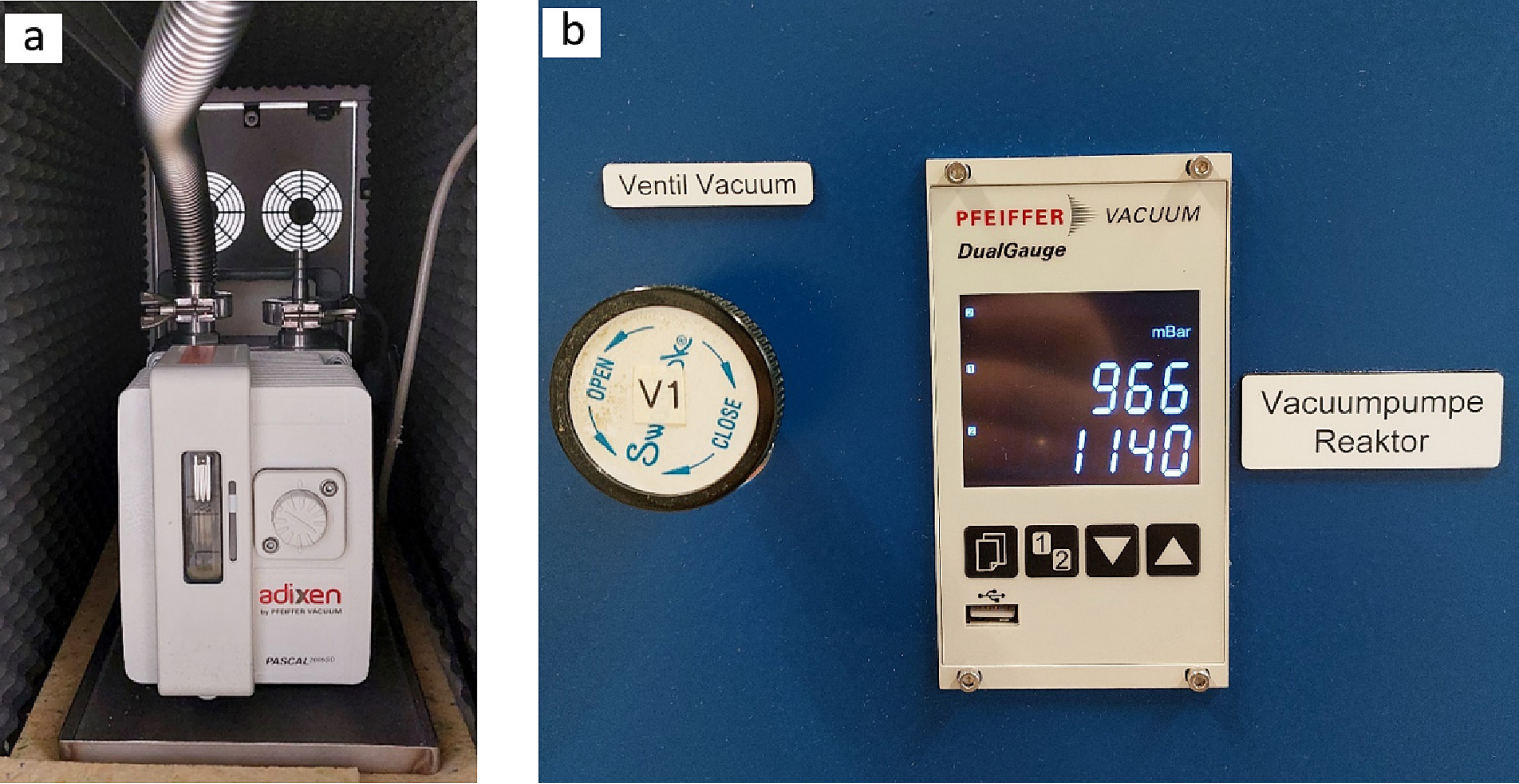
a) Vacuum pump and b) pressure monitoring gauge.

When performing a CO_2_ photoreduction experiment in batch mode, with every sampling event a volume of the reactants and the products is removed from the reaction chamber (more details about the operation of the photoreactor and the experimental protocol can be found in paragraph 6). To ensure that there is enough volume left to collect a series of samples from a single experiment, the pipe connecting the outlet of valve R3 with the GC must be as thin and short as possible. This will allow for a smaller pressure drop resulting in more sampling events to take place. Before each measurement, the sample loop is evacuated using a vacuum pump (adixen Pascal 2005SD, Pfeiffer) equipped with an oil-trap, further reducing the risk of contamination from left-over species. The pressure of the vacuum pump is being monitored by a vacuum gauge meter (TPG 362, Pfeiffer) connected to the PCR 280 (Pfeiffer) controller (Fig. 14).

**Fig. 15:**
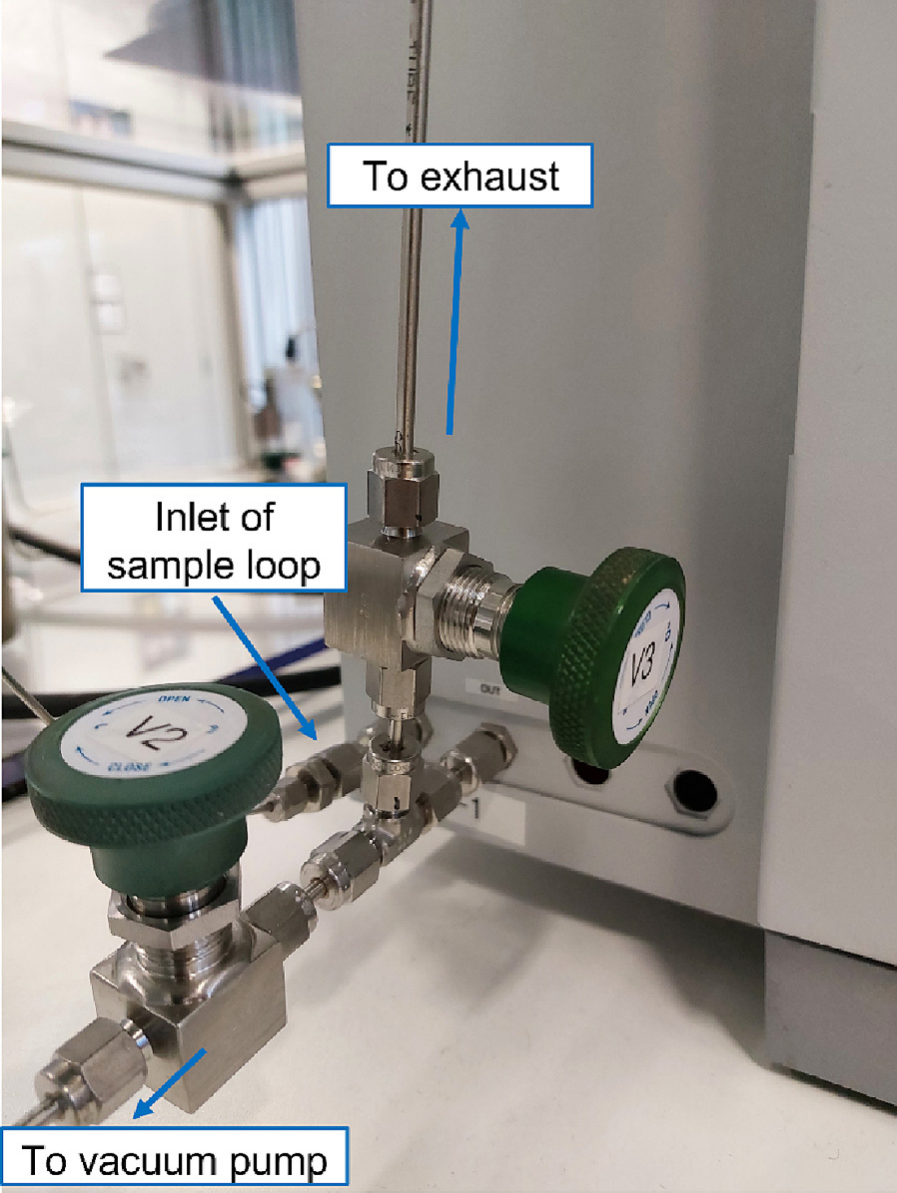
Set of valves attached to the sample port of the Tracera-2010 GC.

With valve V2 open and R3 and V3 closed (Fig. 2, Fig. 15) an evacuation of the outlet of the reactor and the sample loop of the GC is performed. To stop the operation of the vacuum pump, (with valve R3 closed) both V2 and V3 valves should be opened. Then the operation of the vacuum pump can be terminated without any risk of back-diffusion of oil or oil fumes towards the reactor. When no vacuum is needed (e.g., when performing experiments in a flow mode) valve V2 must stay closed while valves R1, R3 and V3 must be open. This allows for the gas to flow continuously through the reactor, the sample loop of the GC and end-up in the exhaust of the laboratory. Periodically, a sample of the flowing gas mixture can be collected by the GC for analysis.

**Fig. 16:**
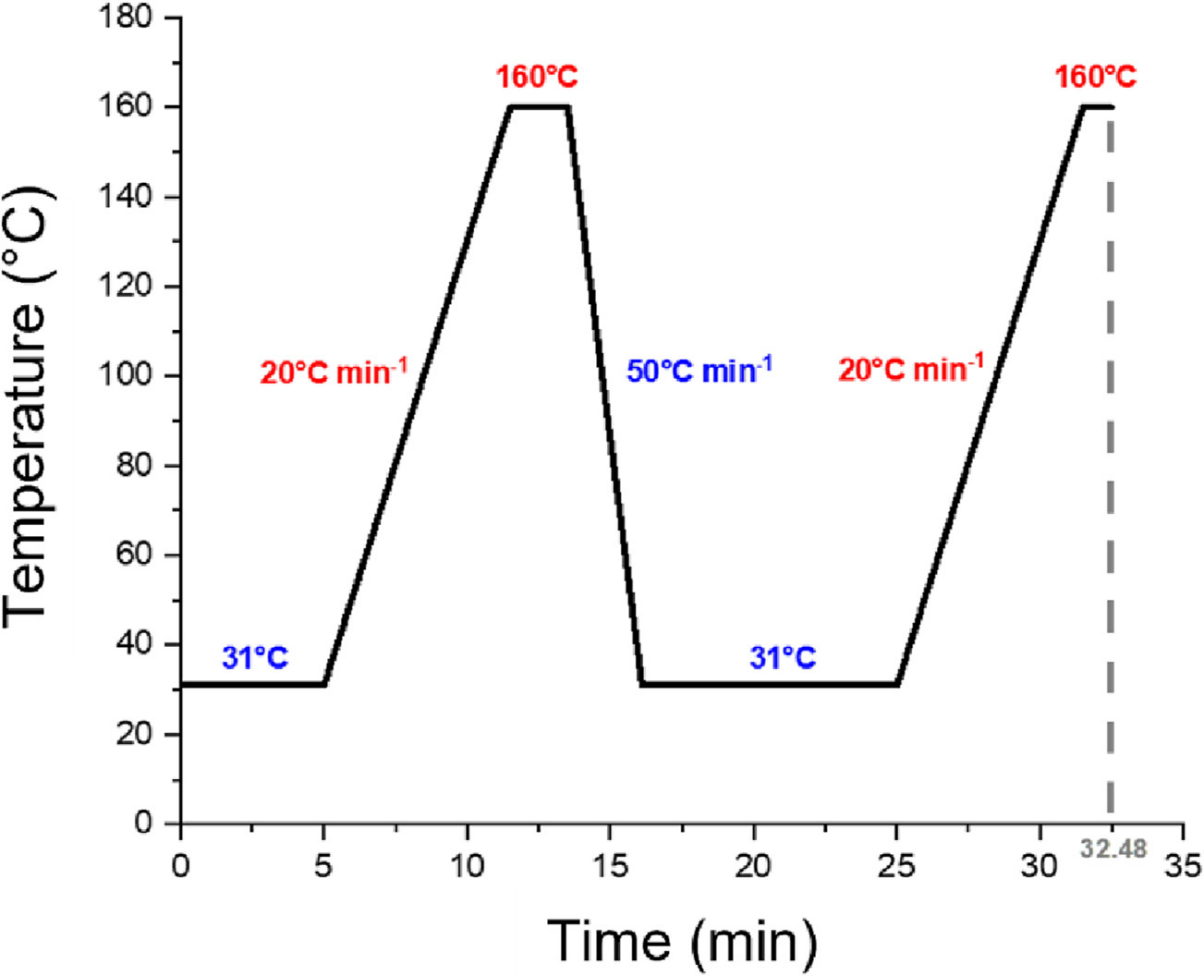
GC temperature profile when measuring the products of the photocatalytic CO_2_ reduction experiments. Blue (red) color indicates low (high) temperatures and cooling (heating) rates.

The difference in the pressure between the reactor (in a batch experiment the pressure range is 500–1500 mbar) and the evacuated sampling unit (typically in the 2 × 10^−3^ mbar range) is driving the sample collection. A full chromatogram is being collected over 32.5 min. A sufficient time is needed for the GC to cool down between measurements. In a CO_2_ reduction experiment, chromatograms are collected every 45 min using the temperature profile presented in Fig. 16.

**Fig. 17:**
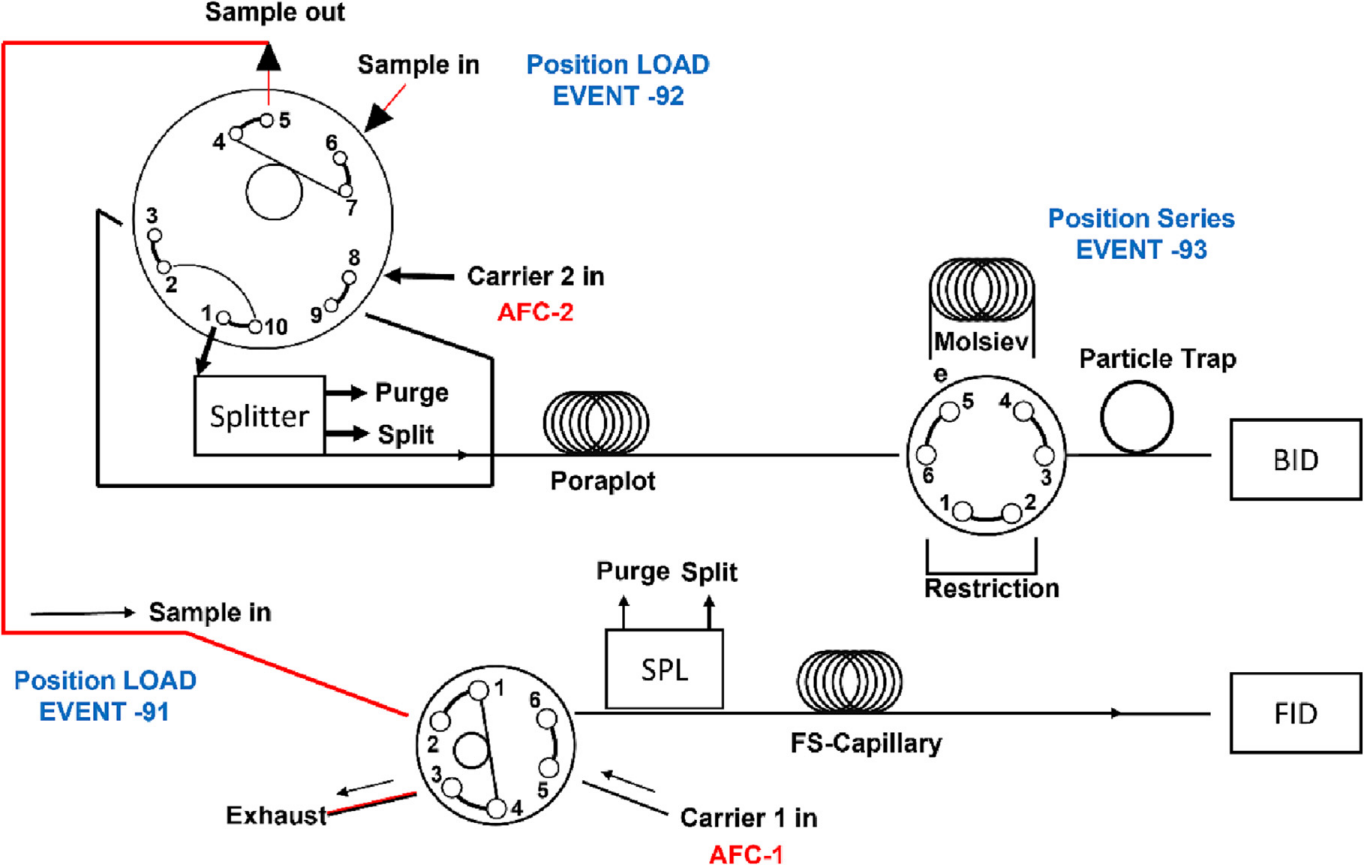
Column setting and sampling procedure in the Shimadzu TRACERA GC.

A detailed schematic of the column setting, and the sample loop can be seen in Fig. 17. Briefly, in a typical sequence, both BID and FID sample loops are filled with the sample collected from the reactor due to the pressure difference towards the sampling unit. The sample loops are flushed with the carrier gas and in the case of the BID the sample is inserted into the Poraplot column used to separate lightweight molecules and permanent gases. As CO, CH_4_ and H_2_ have low interaction with the Poraplot column they are stored in the Molsieve column while the remaining gases (e.g., CO_2_, H_2_O, N_2_, O_2_) are being separated by the Poraplot. Then the stored gases are released and reach the BID detector. A restrictor is used to enable continuous gas flow properties. For the FID, the molecules of the collected sample pass through the FS-Capillary column where hydrocarbons can be identified.

#### 5.2.6 Calibration of the gas chromatograph

**Fig. 18:**
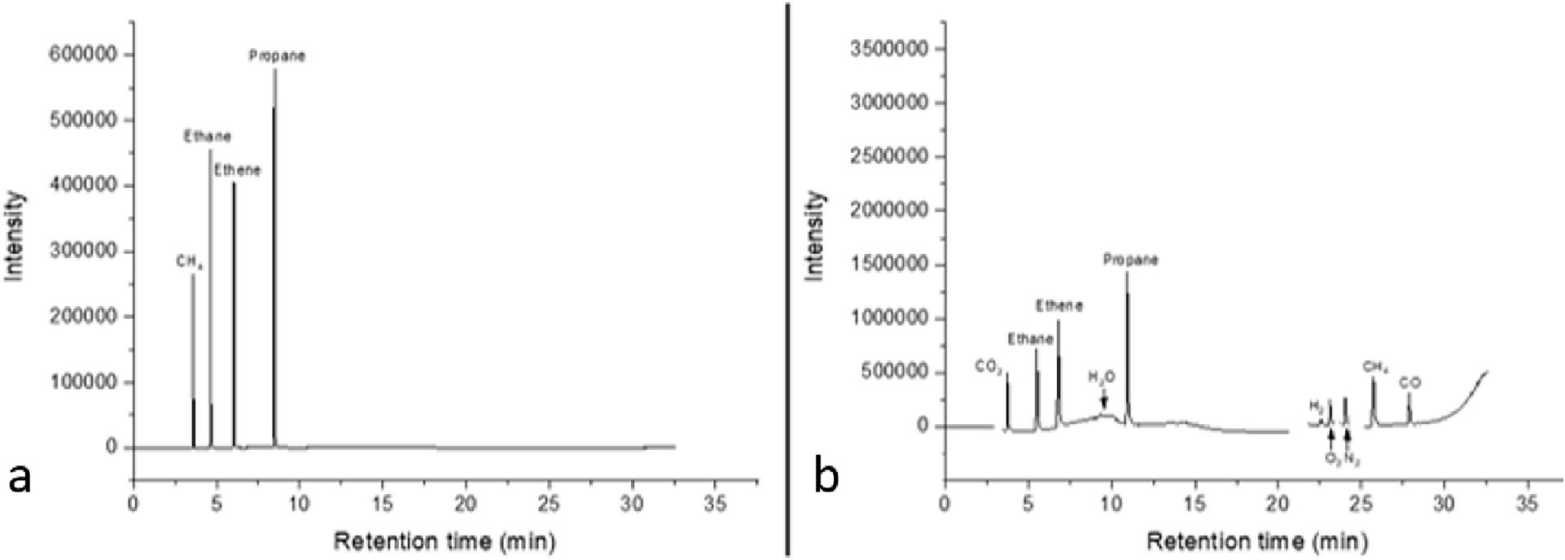
(a) FID and (b) BID chromatograms with the retention times of tested gas molecules.

In the photoreactor presented here, gas chromatography is used for the qualitative and quantitative analysis of the reaction gas atmosphere. To perform qualitative and quantitative analysis of the products of the photocatalytic CO_2_ reduction reaction, the calibration of the GC is necessary. Various concentrations (diluted with 6.0 He) of the main gaseous reactants (CO_2_ and H_2_O) and possible products were measured using the GC. The calibration gas cylinders were connected to MFC B and a continuous flow of the selected gas mixture with pure He from MFC C (Fig. 2) for specific dilution(s) was flushed through the reactor until a steady state of gas phase composition was reached. Then a GC analysis of the actual gas phase was conducted, and the retention time of each molecule was checked for qualitative analysis. The integrated peak area in the resulting chromatogram was then related to the adjusted concentration in the gas flow. Five consecutive chromatograms were collected for every concentration. The area was determined through integration of the respective peaks and the resulting average value was used for the calibration graph. Linear fitting was performed for each gas of interest. For quantitative analysis, the gradient of this slope was used as the calibration factor, allowing to convert the integrated peak area of the specific molecule to its concentration in the gas flow. As an example, the calibration for CH_4_ (often the main product of CO_2_ photoreduction) was performed using ten different concentrations (10, 25, 50, 250, 500, 1000, 2500, 5000 and 10000 ppm). For each of the ten concentrations, five chromatograms were collected and averaged to calculate the calibration curve and equation. The standard deviation was calculated to be ±0.5%. Fig. 18 presents an example of an FID (a) and BID (b) chromatograms where the retention times of the gases used for calibration can be seen.

**Fig. 19:**
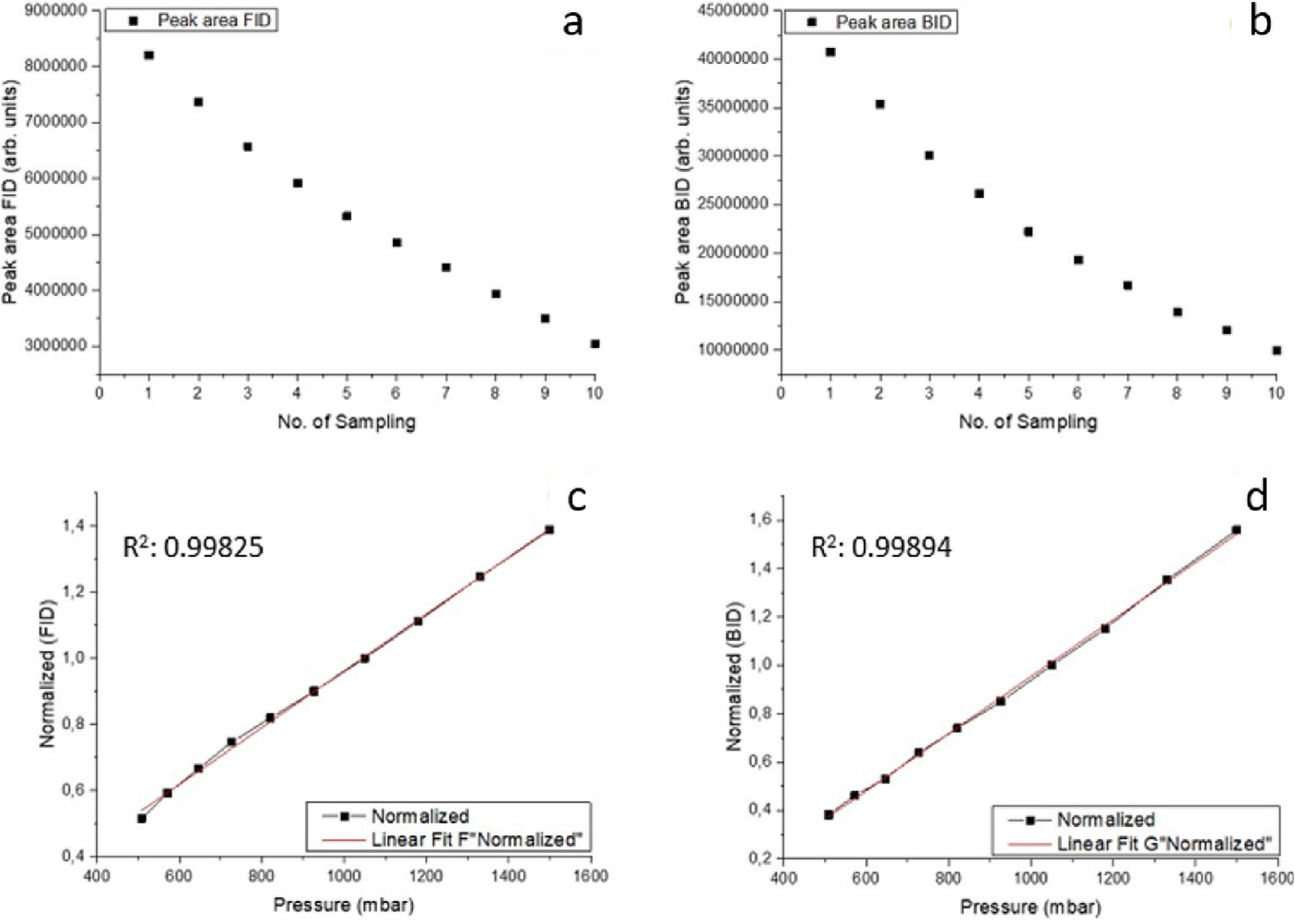
Top row: Peak area against number of samplings for a) FID and b) BID. Bottom row: Normalized area against pressure in the reaction chamber for c) FID and d) BID.

As the pressure inside the reactor decreases with each sampling event in a batch process, the amount of each molecular species and its resulting peak area becomes lower. For this reason, the peak area of each molecule of interest is corrected for the respective pressure drop. A mathematical expression which relates the sample amount to the pressure inside the reactor needs to be derived for compensation. This mathematical expression was determined in a series of GC samplings, as exemplified for CH_4_in the following. The reactor was purged with 1% CH_4_ in He. After 1 h of purging the reactor outlet was closed and the pressure inside the reaction chamber was increased to 1500 mbar. In the following ten gas chromatograms were collected each time removing part of the gaseous atmosphere from the reactor. The peak area against the number of sampling events is plotted for both the FID (Fig. 19a) and BID (Fig. 19b).

From Fig. 19 it can be clearly seen that the peak area changes over the number of GC measurements. The peak areas were normalized over the area of the atmospheric pressure (1020 mbar) measurement and plotted against the pressure inside the reactor (Fig. 19c for FID and Fig. 19d for BID). The resulting linear fitting equations are used to express the product amount as a function of the pressure inside the reaction chamber. Similar correlations should be obtained for all products of interest.

## 6 Operation of the photoreactor in a typical CO_2_ photoreduction experiment

### 6.1 Sample preparation and placement

In a CO_2_ photoreduction experiment it is very important to verify the origin of the identified reaction products. As mentioned before, the presence of C-containing impurities can severely influence the reliability of the results as hydrocarbons may originate from the interaction of the impurities with the photocatalyst and the light and not from the ability of the photocatalyst to reduce CO_2_. The design of the described photoreactor ensures that all potential sources of such construction-related impurities are dealt with. But impurities can also come from the photocatalyst itself from e.g., leftover solvents from synthesis or from incomplete calcination or from possible prior experiments with ethanol. For this reason, it is crucial to perform blank experiments to ensure that without the presence of CO_2_, no products are formed. These blank experiments act also as a "cleaning" step [26]. By introducing only pure He and H_2_O to the reaction chamber under light irradiation, any impurity adsorbed on the surface of the photocatalyst will be removed in the form of hydrocarbon species. With enough time, eventually the photocatalyst surface will be clean of impurities and then CO_2_ can be introduced to the reaction chamber for a CO_2_ reduction experiment to take place. This photo-cleaning method is sufficient to indirectly prove that the products formed originate from the interaction of CO_2_ with the tested photocatalyst. Performing experiments using ^13^C-labelled CO_2_ may be used for further verification [3]. This method, though, is cost intensive as it also requires a mass spectrometer.

The cleaning process can be accelerated by thermally treating the photocatalyst before its introduction into the photoreactor. A suitable temperature must be selected to avoid structurally damaging the photocatalyst. As an example, in case of a pure TiO_2_ photocatalyst (e.g., P25, Evonik) a thermal treatment at 400 °C for 3 h at a rate of 5 °C min^−1^ in a tube furnace in a synthetic air (80% Ν_2_, 20% Ο_2_) environment is suggested. After cooling down to ambient temperature, the sample is introduced into the reactor spread on the quartz sample holder (paragraph 5.2.3). In a typical experiment involving the catalyst in a powder form a mass of 50 mg is used. Gloves must be constantly worn while handling the photocatalyst, the sample holder, and the inside of the reaction chamber as oily fingerprints may leave residues inside the reaction chamber leading to false products. In addition, only water and heat must be used to clean the sample holders as organic solvents might remain adsorbed.

### 6.2 Operation of reactor during photo-cleaning and CO_2_ photoreduction

As the initial heat-treatment might not be sufficient for the complete removal of the C-containing impurities, a second cleaning step is employed as described above ("photo-cleaning"), which takes place with the sample located inside the reactor. This additional cleaning process is carried out at ambient temperature either under continuous flow (flow-through cleaning) or under static conditions (batch cleaning). In a typical flow-through cleaning, 20 ml min^−1^ of pure 6.0 He from MFC C (valves M2 and M4 are open, valves M1 and M3 closed) are continuously flowing through the H_2_O saturator (valves S3 and S4 open, S1 and S2 closed) cooled down at 5 °C (concentration of H_2_O ∼6000 ppm at a pressure of 1020 mbar). This humidified gas feed is flowing over the sample and out to the exhaust (valves R1 and R2 open, R3 closed) under simultaneous irradiation. This allows for the fast removal of the adsorbed impurities from the photocatalyst.

To better monitor the evolution of the products formed from the adsorbed impurities a batch cleaning process is followed where the reactor is filled with the same composition of a He/H_2_O mixture compared to the flow cleaning. The humidified He mixture flows (valves M2, M4, S3 and S4 open, valves M1, S1, S2 and R2 closed) over the sample and through the GC sample loop (valve V2 closed and V3 open). Chromatograms must be collected to ensure that no O_2_ is present. O_2_ plays a detrimental role in CO_2_ photoreduction as it can cause the oxidation of the formed products back to CO_2_ leading to poor performance of the tested photocatalysts. When no O_2_ can be observed, valves V3 and R3 are closed, and the reaction chamber starts filling with the He/H_2_O mixture. When the pressure of the reaction chamber reaches 1500 mbar, valve R1 must be closed and the He flow must be stopped from MFC C to avoid a pressure build-up in the gas-carrying tubes. With the reactor filled and with valves V3 and R3 closed and V1 valve open, the vacuum pump can be switched on. By switching on the pump, the GC sample loop is evacuated allowing for the collection of a sample from the reaction chamber. The targeted pressure is in the range of 2 · 10^−3^ mbar and it can be monitored through the PCR 280 (Pfeiffer) pressure controller. When the pressure reaches the targeted value and to collect a gas sample from the reactor, valve V2 should be closed. Then by opening valve R3 (always with valve V2 closed) and because of the pressure difference between the evacuated GC sample loop and the reaction chamber a gas sample will fill the sample loop. With the sample loop filled, valve R3 must be closed, and a chromatogram can be recorded for the collected sample. After the first measurement has been collected, light irradiation can be initiated. The first measurement taken without light irradiation acts as a baseline and ensures that no O_2_ is present in the reaction chamber. All remaining measurements are taken under illumination. It should not be forgotten that the cooling of the light irradiation sources must be activated (water cooling of the IR filter for the Hg/Xe lamp or the attached cooling fan in the case of the high-power LED) to avoid overheating.

The collection of samples is performed every 45 min (can vary depending on the user's selected product identification technique) with the same procedure as described before: i) With valves R3 and V3 closed, valve V2 is opened to evacuate the GC sample loop, ii) when the desired vacuum is achieved, with valve V2 closed, valve R3 opens and a gas sample is loaded in the sample loop of the GC, iii) valve R3 closes and the gas sample analysis can start. In each measurement, the initial (before opening valve R3) and final (after opening valve R3) pressure values must be noted down as they will be used for the pressure correction as described in paragraph 5.2.6. In total nine data points are collected with the first being without light irradiation present. The final pressure after the collection of the ninth sample is around 500 mbar. As mentioned before, to ensure that the gas sample removed from each sampling event is sufficiently small (as evidenced by the drop of pressure every time that a gas sample fills the sample loop), the distance between valve R3 and the inlet of the sampling port of the GC must be as short as possible and the connecting tube must have a small diameter. In the reactor design presented here, the length and the diameter of the connecting tube is approximately 45 cm and 1/16 in. respectively. In a photocatalytic CO_2_ reduction experiment, the same process is being used as in the batch cleaning step with the only difference being that CO_2_ is added to the system (pure or diluted).

### 6.3 End of experimental procedure

When the final gas sample is collected, the light irradiation can be switched off. Enough time should be allowed for the lamp to cool down before switching off its power unit. While cooling down, a fan located at the side of the lamp housing unit is rotating. When the fan stops, the lamp is sufficiently cooled down and the power unit can be switched off. At this point also the water cooling for the IR filter can be stopped. Before turning off the cryostat used to cool down the H_2_O in the saturators, its pump must be stopped before shutting down the power unit. To switch off the vacuum pump first valve V2 should be opened (always with valve R3 closed). This will clean the sample loop from any remaining sample products. Then valve V3 should be opened and subsequently the vacuum pump can be shut down. With this process, a back-diffusion of oil fumes towards the photoreactor is avoided. At the end of the CO_2_ reduction experiment the tested sample can be removed from the reactor. To do so, the reactor should first be flushed with pure He (valves M2, M4, R1 and R2 open, M1 and R3 closed) and/or H_2_O (valves S3 and S4 open, S1 and S2 closed) to bring the pressure of the reactor back to 1020 mbar. Then the reactor lid can be opened, and the sample can be removed from the reaction chamber. Before putting a new sample inside, the reactor and the water saturators should be flushed with He to remove any left-over CO_2_ thus avoiding contamination of the new sample.

### 6.4 Important precautions to be considered

A list of the most important precautions needed to be taken by the potential user can be found below. These precautions cover both the assembly of the high-purity photoreactor as well as its operation. For a better comprehension of these precautions by the reader, the list has been split into two parts: i) assembly and ii) operation of the photoreactor.

#### 6.4.1 Assembly of the photoreactor

• Users should take every precaution measure possible when handling gases, gas cylinders and pressure regulator. If the persons assembling the photoreactor is not familiar with gas handling, they should reach to a trained professional who can safely perform the necessary gas connections.
• Gloves should be used when assembling the photoreactor to avoid the accumulation of C-containing impurities on the individual parts through deposition of oily residues from the fingertips.
• The appropriate pressure regulators and fittings must be used to ensure a good sealing of the gas cylinders. This will ensure a high-purity environment for the experiments but also help avoiding contamination of the gases in the cylinders by back diffusion. As aforementioned (paragraph 5.2.1), gas cylinders must be safely placed and stored to avoid any danger (e.g., falling of a gas cylinder) for the users.
• Extensive leak tests must be performed while assembling or before operating the photoreactor. Leak tests serve a double purpose: i) to ensure that measurements are performed under high-purity conditions and ii) that no harmful or dangerous gases are escaping to the atmosphere of the laboratory (e.g., CO).
• Potential users must always follow the guidelines and recommendations of the manufacturers of the parts and tools used in the construction and operation of the proposed high-purity photoreactor. Special care should be taken not to overtighten the connecting adaptors as this might lead to leaks.
• All necessary electrical connections (e.g., connection of the heat tapes), must be performed and supervised by a trained professional.

#### 6.4.2 Operation of the photoreactor

• All unnecessary gas flows must be stopped if the respective gases are not needed for an experiment. For example, when the required pressure is reached inside the reaction chamber (batch CO_2_ photoreaction experiment), valve R1 should be closed and any unnecessary gas flow (e.g., He or CO_2_) must be stopped. This is critical to avoid a pressure build-up in the gas-carrying lines. If for any reason (e.g., by forgetting a gas flow) a pressure built-up occurs, the by-pass of the reactor should be used (valves R1, V2 and V3 closed and B1 open) to release the pressure. This is even more critical if the (water) saturators are under high pressure which will possibly result in flooding of the reaction chamber if the pressure is released not through the by-pass but through the reactor (if valve R1 is open). If a powder sample is placed in the reaction chamber, the releasing of the pressure through valve R1 might lead to an accumulation of nanoparticles in the valves which in turn will lead to an imperfect sealing. In any case, it is advised that the opening of valves should be performed slowly while simultaneously monitoring the pressure gauges to ensure that the pressure remains within values safe for the operation of the reactor.
• When the vacuum pump is in operation, valves R3 and V2 must not be open simultaneously as this will result in the evacuation of the reaction chamber and possibly lead to the removal of the sample towards the GC (or any other product identification method).
• The connections of the water circulation piping system should be regularly checked, as any water spillage might result in a short circuit damaging the equipment. In cases where the temperature in the laboratory is high, water condensation might occur in the outer surface of the saturators and the water-carrying pipes so special attention should be paid that no water accumulates close to electricity-powered equipment.
• Gloves should be always used when handling the reaction chamber as oily residues from the fingertips can accumulate in the metallic surfaces potentially producing carbon-containing molecules under light irradiation.
• When the irradiation source is in operation (e.g., Hg/Xe lamp) it must be ensured that no light escapes from the setup as this might lead to damaged eyesight and skin burns. Special glasses should be worn in any case when light might escape during operation.
• When operating the Hg/Xe lamp cooling water must flow through the attached IR-filter. Otherwise, the increased temperature might evaporate the water and break the filter. Once the Hg-Xe lamp is turned on, it should be kept in operation for 2–3 h before switching it off. Turning it on and off at short time intervals will degrade the light bulb and reduce its lifetime. When turning off the lamp (e.g., at the end of an experiment), it should be allowed to cool down (the big fan at the side of the lamp housing will stop spinning) before switching off the power unit.
• If the vacuum pump needs to be stopped, valves V2 and V3 must be opened first before stopping its operation. Otherwise, a diffusion of oil (or oil fumes) from the pump might occur towards the GC and the reaction chamber.

## 7 Validation of reaction products

Apart from metal oxides, various families of photocatalysts are studied in photocatalytic CO_2_ reduction, including graphitic carbon nitride-based photocatalysts (g-C_3_N_4_) [27], [28], metal–organic frameworks (MOFs) [29], [30], and Z-scheme structures [31]. When it comes to metal oxides in heterogeneous photocatalytic CO_2_ reduction, titanium dioxide (TiO_2_) is the most often used metal oxide photocatalyst either as the main studied material (pure or modified) or as a benchmark material for comparison purposes [32]. Among all commercially available TiO_2_ photocatalysts, P25 is considered to be the catalyst-of-choice. The coexistence of two TiO_2_ polymorphs (80% anatase – 20% rutile) in it is considered beneficial for CO_2_ photoreduction (and photocatalysis in general) as it facilitates the separation of the photogenerated charge carriers (electrons -e^−^- and holes -h^+^-) [33].

**Fig. 20:**
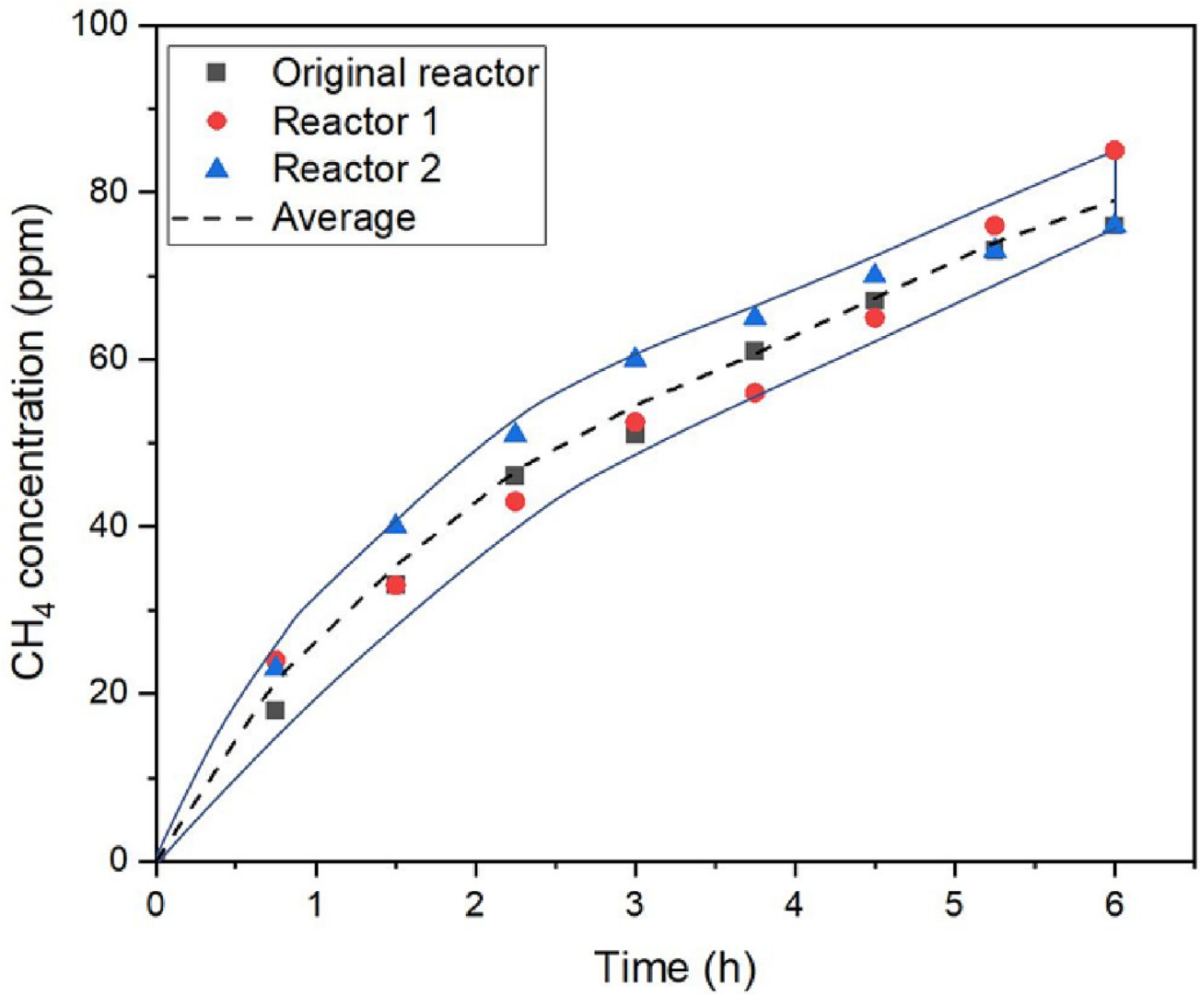
Production of CH_4_ using the original and the optimized designs of the high purity photoreactor. Experimental conditions: 1.5% CO_2_ in pure He, 6000 ppm H_2_O, 200 W Hg/Xe lamp. For the original design the results are included in [19].

As mentioned before, the photoreactor described in detail in this work is an evolution of a high-purity gas-phase photoreactor setup constructed and operated by our respective groups in the past. As a measure of reproducibility of the results between the original and two interpretations of the evolved design of the high-purity photoreactors (namely, Reactor 1 and Reactor 2), batch CO_2_ photoreduction experiments were performed in all of them under similar experimental conditions (1.5% CO_2_ – 15,000 ppm) in pure He, 6000 ppm H_2_O, 200 W Hg/Xe lamp) by two different users. The results are presented in Fig. 20. The CO_2_:H_2_O concentration ratio was selected based on previous studies of our groups which showed that lower (than pure) concentration of CO_2_ and H_2_O seem to be beneficial in the case of P25.

As it can be seen the results of the photoreduction of CO_2_ on TiO_2_ P25 in the three reactors – constructed and operated by different operators in different laboratories – display very similar product yields in terms of CH_4_ production thus ensuring a high level of reproducibility of results among the two reactor designs (CH_4_ concentration: 79.0 ± 4.2 ppm after 6 h). Minor differences in product formation are expected as spreading the photocatalyst onto the sample holder introduces a negligible randomness factor to the experiment. Traces of ethane (<3 ppm) and ethene (<1 ppm) were also produced during the experiments.

Measurement errors associated with the presented results can be categorized as either equipment-related or user-induced. In our photoreactor design, the electronic equipment-related errors are the following:

i. Mass flow controller (MFC): 1.2 % (0.5% RD-Percentage of reading- and 0.1 % FS -Percentage of full scale-, e.g., 10 ml ± 0.12 ml min^−1^, values from the manufacturer's website).
ii. Pressure gauge: 0.1 % (value from the manufacturer's website).
iii. Gas chromatograph (GC): 0.5% (calculated through the GC calibration – see section 5.2.6)

As all measurements presented in Fig. 20 were performed in batch mode (no continuous flow), the error induced by the MFCs can be potentially excluded. The error of the measurement depends on the equipment that the potential user selects.

When the measurement process described in the manuscript is followed carefully, the margin of user-induced error is very narrow as during the measurements the operator should open / close two valves. A variation factor exists related to the distribution of the photocatalytic powder onto the quartz sample holder. A certain degree of randomness is introduced, as the spreading of the powder cannot be exactly replicated by the same or by different users. An additional error-inducing factor is material related: P25 has an inconsistent crystalline composition in the same batch [34]. The anatase:rutile ratio of P25 is often referenced in literature to be 70:30 or 80:20 but in many cases some amorphous material is also present. This inconsistency can also be indirectly inferred by the supplying company as in the BET area a range of 35–65 m^2^ g^−1^. Based on the above, the deviation observed in the measurements presented in Fig. 20, can very likely originate from material-related factors and not from the photoreactor design itself [35].

A direct literature comparison of P25′s performance in CH_4_ formation from CO_2_ is unfortunately not possible because of the lack of reactor and measurement standardization. The DIN SPEC 91457 protocol is aiming at providing such a standardization. The photoreactor design and measuring protocol are in accordance with this national standard [36]. From the publication of Kondratenko et al. it can be seen that (without accounting for high-purity conditions) regardless of the photoreactor and experimental parameters used in the respective articles, the average referenced efficiencies for CH_4_ production from TiO_2_-based materials is around 10 μmol g_cat_^−1^ h^−1^ [2]. An ongoing study by our groups indicates that this average efficiency (again without focusing on high-purity conditions) increased to 20 μmol g_cat_^−1^ h^−1^ in the past ten years. In the experiments presented in Fig. 20, where all the possible C-impurities sources are taken into consideration, the concentration of CH_4_ produced (around 80 ppm in six hours) by an unmodified P25 is translated to approximately 0.4 μmol g_cat_^−1^ h^−1^ under the selected experimental conditions.

## 8 Conclusions

In this work, a detailed description of the design and construction of a high-purity gas-phase photoreactor setup has been described. In addition, a thorough guide of the operation procedure of the reactor based on an experimental protocol for the photocatalytic reduction of CO_2_ is provided. By following the described construction and operation steps included in this work, the potential user will be able to collect accurate and reproducible experimental data, not only in CO_2_ photoreduction but also in other (light-driven) gas–solid reactions requiring mild temperature and pressure.

## Declaration of Competing Interest

The authors declare that they have no known competing financial interests or personal relationships that could have appeared to influence the work reported in this paper.

## References

[1] U.S.D.o.C. National Oceanic and Atmospheric Administration. Vital Signs of the Planet: Carbon Dioxide. Available online: https://climate.nasa.gov/vital-signs/carbon-dioxide/ (accessed on 20/4/2023).

[2] Kondratenko E.V., Mul G., Baltrusaitis J., Larrazábal G.O., Pérez-Ramírez J. (2013). Status and perspectives of CO2 conversion into fuels and chemicals by catalytic, photocatalytic and electrocatalytic processes. Energy Environ. Sci..

[3] Moustakas N.G., Strunk J. (2018). Photocatalytic CO2 reduction on TiO2 -based materials under controlled reaction conditions: systematic insights from a literature study. Chem. Eur. J..

[4] Wang H.-N., Zou Y.-H., Sun H.-X., Chen Y., Li S.-L., Lan Y.-Q. (2021). Recent progress and perspectives in heterogeneous photocatalytic CO2 reduction through a solid–gas mode. Coord. Chem. Rev..

[5] Habisreutinger S.N., Schmidt-Mende L., Stolarczyk J.K. (2013). Photocatalytic reduction of CO2 on TiO2 and other semiconductors. Angew. Chem.-Int. Edit..

[6] Chang X., Wang T., Gong J. (2016). CO2 photo-reduction: insights into CO2 activation and reaction on surfaces of photocatalysts. Energy Environ. Sci..

[7] Tu W., Zhou Y., Zou Z. (2014). Photocatalytic conversion of CO2 into renewable hydrocarbon fuels: state-of-the-art accomplishment, challenges, and prospects. Adv. Mater..

[8] Li K., Peng B., Peng T. (2016). Recent advances in heterogeneous photocatalytic CO2 conversion to solar fuels. ACS Catal..

[9] Li K., An X., Park K.H., Khraisheh M., Tang J. (2014). A critical review of CO2 photoconversion: catalysts and reactors. Catal. Today.

[10] Ola O., Maroto-Valer M.M. (2015). Review of material design and reactor engineering on TiO2 photocatalysis for CO2 reduction. J. Photochem. Photobiol. C-Photochem. Rev..

[11] Wu J.C.S., Lin H.-M., Lai C.-L. (2005). Photo reduction of CO2 to methanol using optical-fiber photoreactor. Appl. Catal. A.

[12] Nguyen T.-V., Wu J.C.S. (2008). Photoreduction of CO2 to fuels under sunlight using optical-fiber reactor. Sol. Energy Mater. Sol. Cells.

[13] Ola O., Maroto-Valer M.M. (2016). Synthesis, characterization and visible light photocatalytic activity of metal based TiO2 monoliths for CO2 reduction. Chem. Eng. J..

[14] Lo C.-C., Hung C.-H., Yuan C.-S., Wu J.-F. (2007). Photoreduction of carbon dioxide with H2 and H2O over TiO2 and ZrO2 in a circulated photocatalytic reactor. Sol. Energy Mater. Sol. Cells.

[15] Varghese O.K., Paulose M., LaTempa T.J., Grimes C.A. (2009). High-rate solar photocatalytic conversion of CO2 and water vapor to hydrocarbon fuels. Nano Lett..

[16] Freund H.J., Roberts M.W. (1996). Surface chemistry of carbon dioxide. Surf. Sci. Rep..

[17] Yang C.-C., Yu Y.-H., van der Linden B., Wu J.C.S., Mul G. (2010). Artificial photosynthesis over crystalline TiO2-based catalysts: fact or fiction?. J. Am. Chem. Soc..

[18] Mei B., Pougin A., Strunk J. (2013). Influence of photodeposited gold nanoparticles on the photocatalytic activity of titanate species in the reduction of CO2 to hydrocarbons. J. Catal..

[19] Pougin A., Dilla M., Strunk J. (2016). Identification and exclusion of intermediates of photocatalytic CO2 reduction on TiO2 under conditions of highest purity. Phys. Chem. Chem. Phys..

[20] Moustakas N.G., Lorenz F., Dilla M., Peppel T., Strunk J. (2021). Pivotal role of holes in photocatalytic CO2 reduction on TiO2. Chemistry.

[21] Dilla M., Schlögl R., Strunk J. (2017). Photocatalytic CO2 reduction under continuous flow high-purity conditions: quantitative evaluation of CH4 formation in the steady-state. ChemCatChem.

[22] Dilla M., Moustakas N.G., Becerikli A.E., Peppel T., Springer A., Schlogl R., Strunk J., Ristig S. (2019). Judging the feasibility of TiO2 as photocatalyst for chemical energy conversion by quantitative reactivity determinants. Phys. Chem. Chem. Phys..

[23] Dilla M., Mateblowski A., Ristig S., Strunk J. (2017). Photocatalytic CO2 reduction under continuous flow high-purity conditions: influence of light intensity and H2O concentration. ChemCatChem.

[24] Dilla M., Jakubowski A., Ristig S., Strunk J., Schlögl R. (2019). The fate of O2 in photocatalytic CO2 reduction on TiO2 under conditions of highest purity. Phys. Chem. Chem. Phys..

[25] Forero-Cortés P.A., Marx M., Moustakas N.G., Brunner F., Housecroft C.E., Constable E.C., Junge H., Beller M., Strunk J. (2020). Transferring photocatalytic CO2 reduction mediated by Cu(N^N)(P^P)+ complexes from organic solvents into ionic liquid media. Green Chem..

[26] Yang C.-C., Vernimmen J., Meynen V., Cool P., Mul G. (2011). Mechanistic study of hydrocarbon formation in photocatalytic CO2 reduction over Ti-SBA-15. J. Catal..

[27] Cao S., Low J., Yu J., Jaroniec M. (2015). Polymeric photocatalysts based on graphitic carbon nitride. Adv. Mater..

[28] Fu J., Yu J., Jiang C., Cheng B. (2018). g-C3N4-based heterostructured photocatalysts. Adv. Energy Mater..

[29] Zhang T., Lin W. (2014). Metal–organic frameworks for artificial photosynthesis and photocatalysis. Chem. Soc. Rev..

[30] Cui J.-X., Wang L.-J., Feng L., Meng B., Zhou Z.-Y., Su Z.-M., Wang K., Liu S. (2021). A metal-free covalent organic framework as a photocatalyst for CO2 reduction at low CO2 concentration in a gas–solid system. J. Mater. Chem. A.

[31] Low J.X., Jiang C., Cheng B., Wageh S., Al-Ghamdi A.A., Yu J.G. (2017). A review of direct Z-Scheme photocatalysts. Small Methods.

[32] Strunk J. (2018). Metal Oxides in Energy Technologies.

[33] Schneider J., Matsuoka M., Takeuchi M., Zhang J., Horiuchi Y., Anpo M., Bahnemann D.W. (2014). Understanding TiO2 photocatalysis: mechanisms and materials. Chem. Rev..

[34] Takashima M., Ohtani B. (2021). Materials Science in Photocatalysis.

[35] Su R., Bechstein R., Sø L., Vang R.T., Sillassen M., Esbjörnsson B., Palmqvist A., Besenbacher F. (2011). How the anatase-to-rutile ratio influences the photoreactivity of TiO2. J. Phys. Chem. C.

[36] DIN SPEC 91457 Photocatalysis – Determination of product formation in CO2 reduction, https://www.din.de/en/innovation-and-research/din-spec-en/current-din-specs (accessed on 30/6/2023).

