# Supplementary material for: A high-purity gas–solid photoreactor for reliable and reproducible photocatalytic CO2 reduction measurements
Source: HardwareX. 2023 Jun 28;15:e00448. doi: 10.1016/j.ohx.2023.e00448 (PMC10545968; doi:10.1016/j.ohx.2023.e00448)
Supplement: Supplementary data 1 [file mmc1.docx]

**SUPPLEMENTARY INFORMATION**

**A high-purity gas-solid photoreactor for reliable and reproducible photocatalytic CO_2_ reduction measurements**

**Nikolaos G. Moustakas, Marcus Klahn, Bastian T. Mei, Anna Pougin, Martin Dilla, Tim Peppel, Simon Ristig, Jennifer Strunk**


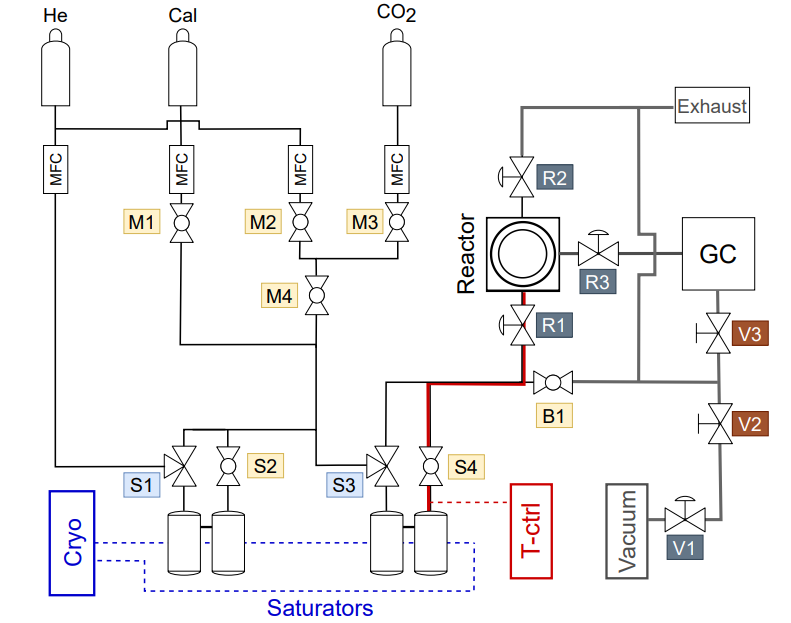


**Figure S1.:** Photoreactor flow chart.


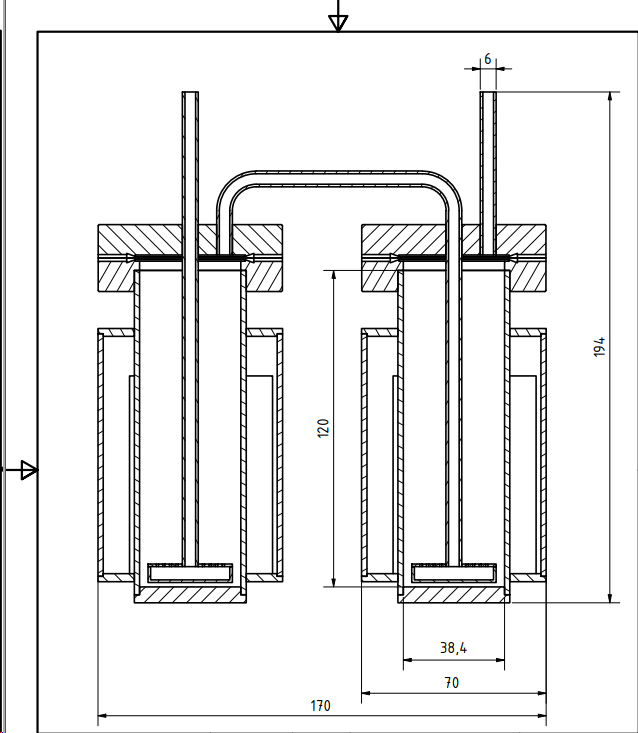


**Figure S2.:** Cross-section and dimensions (in mm) of a water saturator.


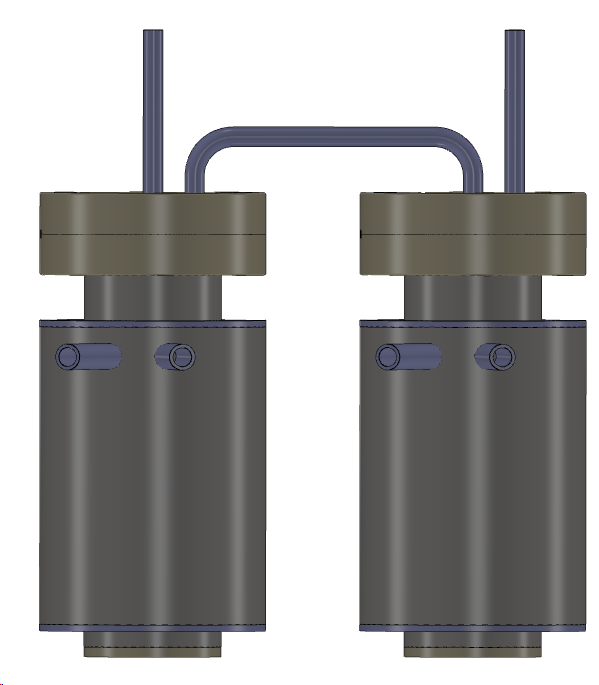


**Figure S3.:** Back-view of the water saturator with the four pipes used to supply (cooling) water (sides of the main body and the gas supply piping system (top side).


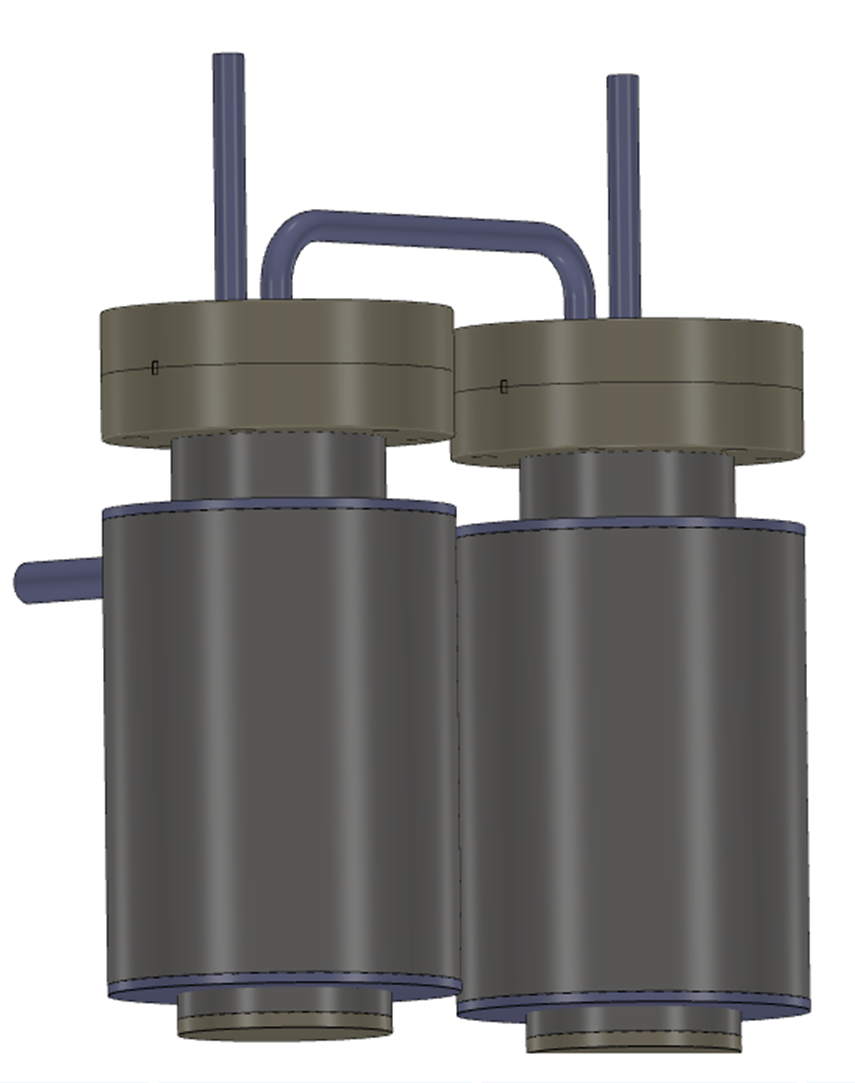


**Figure S4.:** Angled front-view of the water saturator


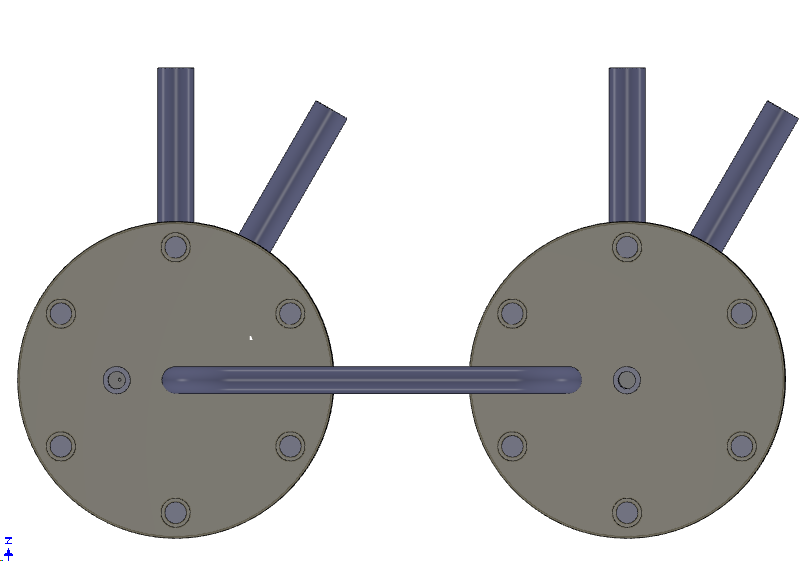


**Figure S5.:** Top-view of the water saturator


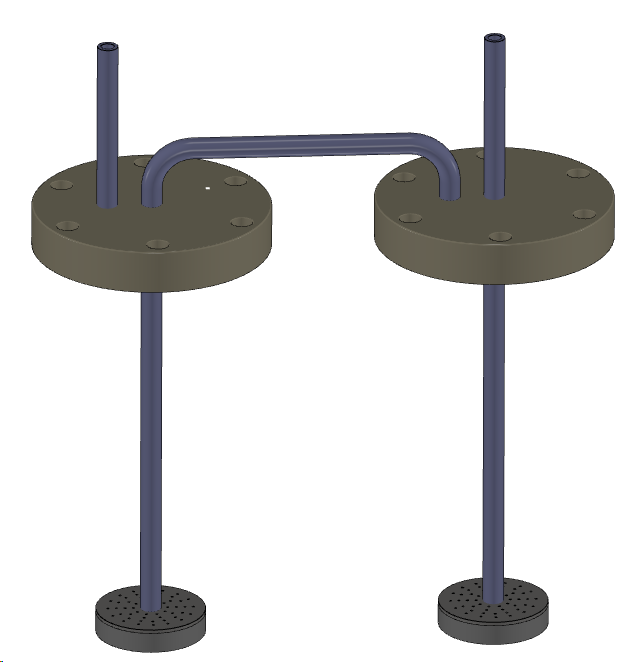


**Figure S6.:** Detached gas-piping system and bubbler


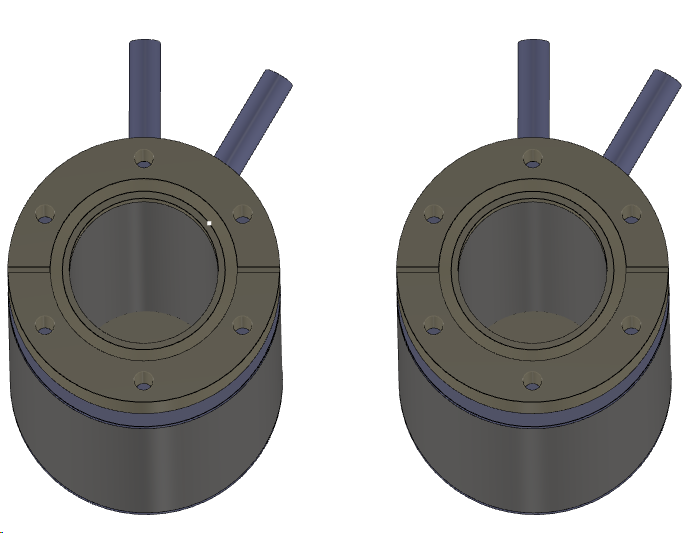


**Figure S7.:** Top-view of the water saturator withouth the gas-piping system and bubbler


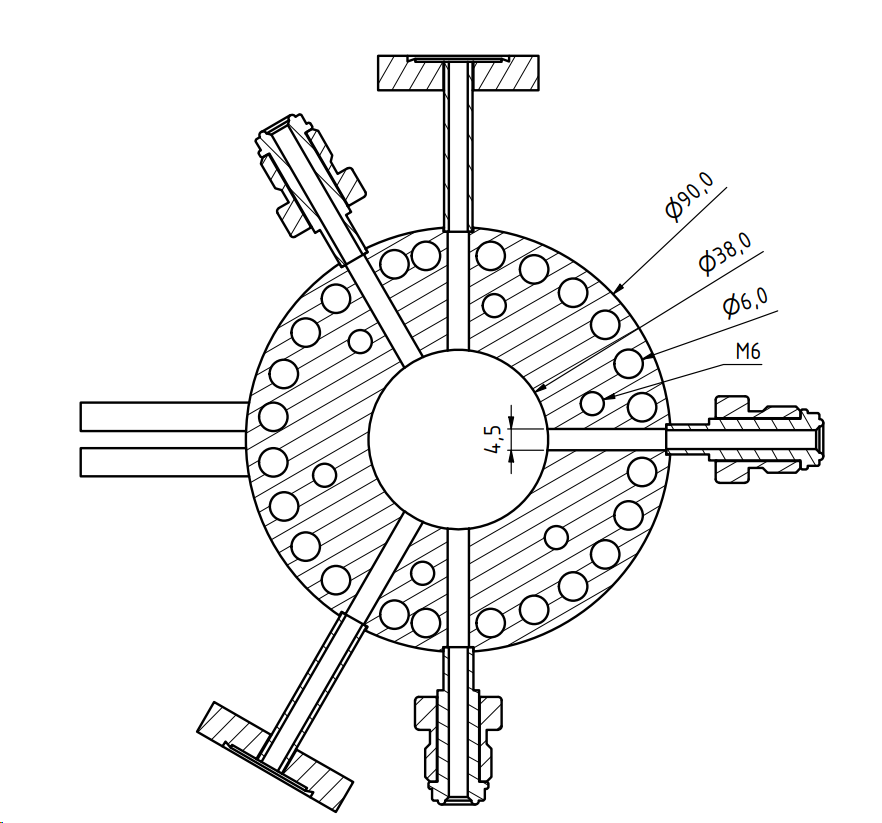


**Figure S8.:** Cross-section and dimensions of the main body of the reaction chambe


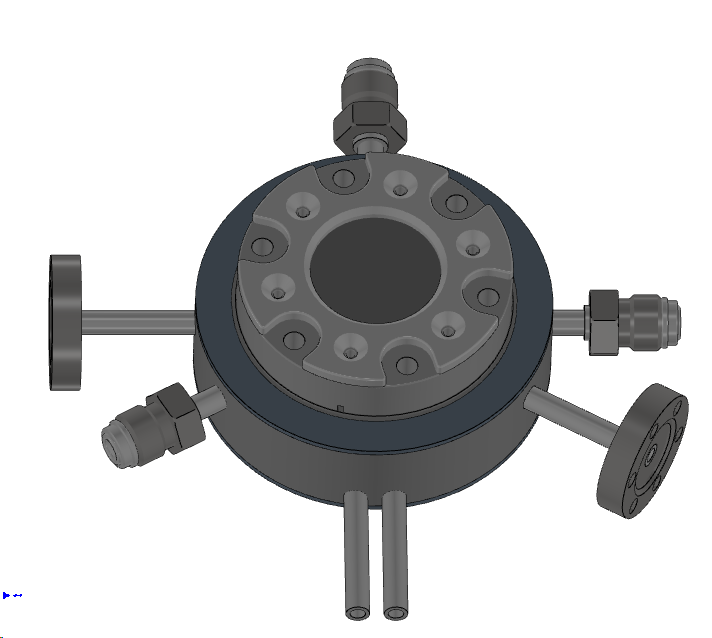


**Figure S9.:** Main body of the photoreactor with the lid attached.


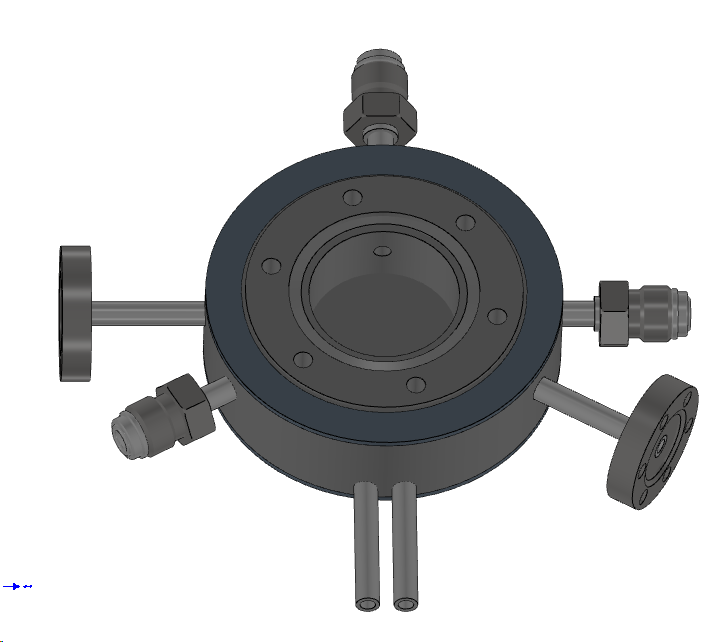


**Figure S10.:** Main body of the photoreactor without the lid.


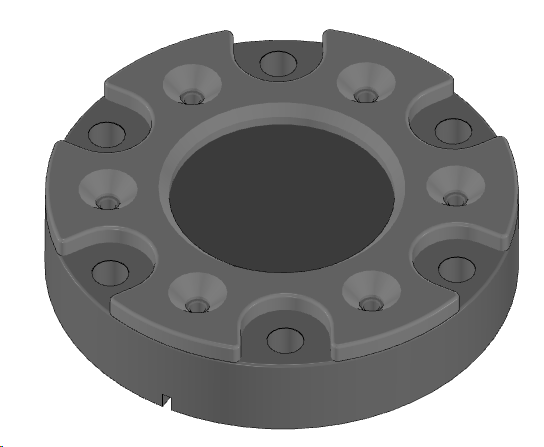


**Figure S11.:** Photoreactor lid (top side).


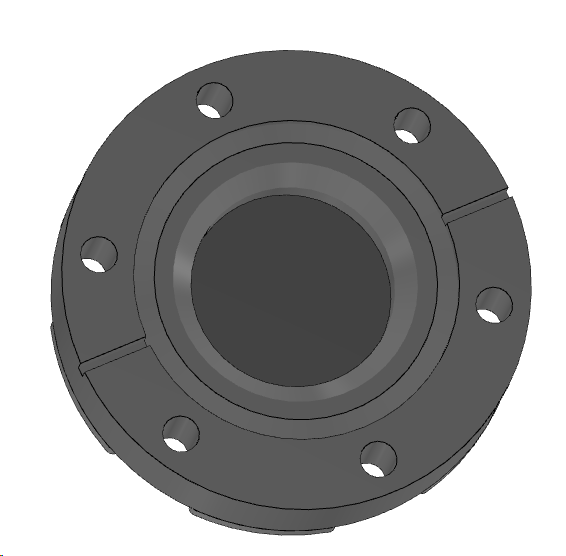


**Figure S12.:** Photoreactor lid (bottom side).
